# The *ACE1* secondary metabolite gene cluster is a pathogenicity factor of wheat blast fungus

**DOI:** 10.1038/s42003-024-06517-7

**Published:** 2024-07-04

**Authors:** Trinh T. P. Vy, Yoshihiro Inoue, Soichiro Asuke, Izumi Chuma, Hitoshi Nakayashiki, Yukio Tosa

**Affiliations:** 1 Graduate School of Agricultural Science, Kobe University, Kobe, 657-8501 Japan; 2 Graduate School of Agriculture, Kyoto University, Kyoto, 606-8502 Japan; 3 Obihiro University of Agriculture and Veterinary Medicine, Obihiro, 080-8555 Japan

**Keywords:** Plant breeding, Fungal pathogenesis

## Abstract

Wheat blast caused by *Pyricularia oryzae* pathotype *Triticum* is now becoming a very serious threat to global food security. Here, we report an essential pathogenicity factor of the wheat blast fungus that is recognized and may be targeted by a rice resistance gene. Map-based cloning of *Pwt2* showed that its functional allele is the *ACE1* secondary metabolite gene cluster of the wheat blast fungus required for its efficient penetration of wheat cell walls. *ACE1* is required for the strong aggressiveness of *Triticum*, *Eleusine*, and *Lolium* pathotypes on their respective hosts, but not for that of *Oryza* and *Setaria* pathotypes on rice and foxtail millet, respectively. All *ACE1* alleles found in wheat blast population are recognized by a rice resistance gene, *Pi33*, when introduced into rice blast isolates. *ACE1* mutations for evading the recognition by *Pi33* do not affect the aggressiveness of the rice blast fungus on rice but inevitably impair the aggressiveness of the wheat blast fungus on wheat. These results suggest that a blast resistance gene already defeated in rice may be revived as a durable resistance gene in wheat by targeting an Achilles heel of the wheat blast fungus.

## Abstract (web summary)

Molecular analyses suggest that a blast resistance gene already defeated in rice may be revived as a durable resistance gene in wheat by targeting ACE1, a pathogenicity factor of the wheat blast fungus.

## Introduction

We are now threatened not only by a human pandemic disease but also by a plant disease that is becoming pandemic^1^. Wheat blast, a destructive disease of our staple crop, first emerged in Brazil in 1985^2^. It subsequently spread to neighboring countries, i.e., Bolivia in 1996, Paraguay in 2002, Argentina in 2007, and became a major threat to wheat production in South America^3^. Its causal agent is a subgroup of *Pyricularia oryzae* (syn. *Magnaporthe oryzae*)^4^, i.e., the *Triticum* pathotype (MoT)^5^ which evolved through host jumps in 1980s^6^. MoT (the wheat blast fungus) spread across continents from South America to Asia and Africa independently^1^, and caused wheat blast outbreaks in Bangladesh (in 2016)^7,8^ and in Zambia (in 2018)^9^. To mitigate this serious disease, resistant cultivars are needed. However, resistance genes effective against wheat blast are rarely found in wheat germplasms probably because, until recently, wheat has had no interaction with MoT. Some resistance genes or resources have been found in wheat and its relatives, e.g., *Rmg8*^10^ in common wheat and a 2NS chromosomal segment derived from *Aegilops ventricosa*^11^. However, the current wheat blast population in South America already contains isolates that have almost overcome the *Rmg8* and 2NS resistance^11–13^.

In the history of breeding for resistance, most of resistance genes introduced into elite varieties have been overcome by new races and rendered ineffective soon after their releases to farmer’s fields. The ultimate goal of plant breeders struggling against plant diseases is to develop cultivars with durable resistance^14^. A big challenge for them is how to predict durability of resistance genes before starting breeding programs^15^. Resistance genes are most likely to be durable if their corresponding virulence is costly^14,16^ or if their corresponding avirulence genes encode Achilles heel effectors essential for virulence^17,18^. It is, therefore, strongly desired to find pathogen genes essential for virulence or pathogenicity and resistance genes targeting their products.

*P. oryzae* is composed of several host genus-specific pathotypes such as *Oryza* pathotype (MoO) pathogenic on rice, *Setaria* pathotype (MoS) pathogenic on foxtail millet, *Eleusine* pathotype (MoE) pathogenic on finger millet, and *Lolium* pathotype (MoL) pathogenic on perennial ryegrass^19,20^, in addition to the newly evolved MoT mentioned above. These pathotypes almost correspond to distinct lineages deduced from phylogenetic analyses^21^. Through genetic analyses involving crosses between wheat (MoT) and non-wheat (MoO and MoS) pathotypes, we have previously identified three loci, *Pwt1*, *Pwt2*, and *Pwt5*, conditioning their specificity on wheat^22,23^. The avirulence/virulence alleles at these loci were designated as *PWT1/pwt1*, *PWT2/pwt2*, and *PWT5/pwt5*, based on a hypothesis that the avirulence alleles were functional^23,24^. Among the three loci, however, *Pwt2* was associated with a peculiar phenotype; under the absence of *PWT1* and *PWT5*, *PWT2* alleles from an MoO isolate (PO12-7301-2) and an MoS isolate (GFSI1-7-2) produced discrete green lesions (designated as G−) while the *pwt2* allele from an MoT isolate (Br48) produced confluent green lesions (designated as G+)^22,23^. The *PWT2* phenotype may be better described as “virulent but weakly aggressive” rather than avirulent. The phenotype produced by carriers of *PWT2* alone was temperature-sensitive; it was indistinguishable from the completely avirulent phenotype at 20 °C, became visible as G− at 22–25 °C, and turned indistinguishable from the completely virulent phenotype at 27–28 °C^22–24^. Cytologically, the discrete lesion with the *PWT2* allele was associated with the higher frequency of papilla formation at penetration sites leading to reduced level of successful host invasion, while the confluent lesion with the *pwt2* allele was associated with the reduced frequency of papilla formation^22,23^. These observations suggest that, regarding the function of *Pwt2*, another hypothesis is possible; the functional allele at the *Pwt2* locus may be *pwt2* (derived from MoT) which actively suppresses the penetration defense (papilla formation) of wheat.

In the present study, we found that the functional allele at the *Pwt2* locus was *pwt2* derived from Br48 (MoT). The *pwt2* allele was a fungal secondary metabolite gene cluster known as the *ACE1* cluster^25,26^. It was involved in the suppression of papilla formation, and essential for the complete pathogenicity with strong aggressiveness of MoT on wheat. In addition, we found that a blast resistance gene, *Pi33*, in rice could recognize *ACE1* alleles derived from MoT and confer resistance. A point mutation inactivating the enzymatic activity of *ACE1*^25^ in MoT for evading the recognition by *Pi33* inevitably caused critical reduction of its aggressiveness on wheat. These results suggest that *ACE1* and *Pi33* may be an Achilles heel of MoT and an arrow targeting it. Its implications in the breeding for durable resistance are discussed here.

## Results

### *Pwt2* is a secondary metabolite gene cluster essential for the strong aggressiveness of MoT on wheat

There are two styles of usage of terms for pathogenicity. In one style virulence is defined as the degree of pathogenicity of a given pathogen^27^, and the term aggressiveness is rejected^28^. In the other style virulence/avirulence is defined as the ability/inability of a pathogen to cause a compatible reaction on a host cultivar with genetic resistance while aggressiveness is defined as the amount of disease caused by an isolate of the pathogen^29^. In the present article, we will use these terms in accordance with the latter usage.

**Fig. 1:**
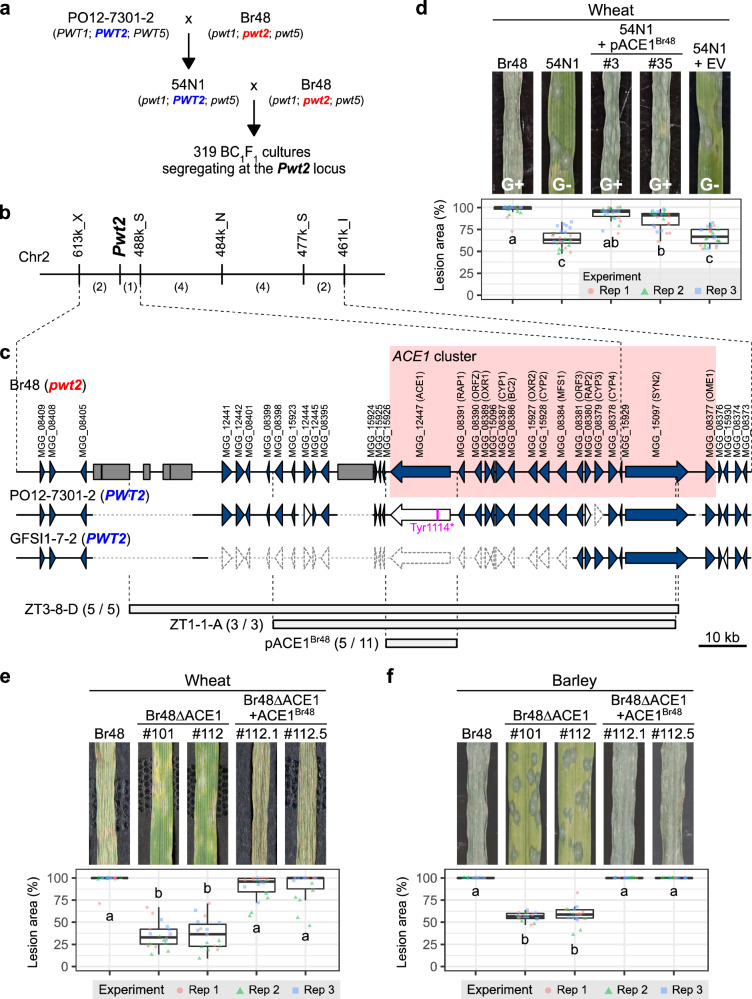
*Pwt2* is a locus conditioning aggressiveness and containing avirulence gene *ACE1.* **a** Production of a BC_1_F_1_ population for mapping of *Pwt2*. **b** Genetic map around the *Pwt2* locus constructed using the BC_1_F_1_ population. Numbers of recombinants are in parentheses. **c** Physical maps of the *Pwt2* locus in Br48 (MoT), PO12-7301-2 (MoO), and GFSI1-7-2 (MoS). Blue arrows/arrowheads, genes; grey boxes, transposable elements; pink vertical line, a nonsense mutation (Tyr1114 to stop); arrows/arrowheads drawn with dotted lines, missing genes; ZT3-8-D/ZT-1-1-A, BAC clones of Br48; pACE1^Br48^, a plasmid clone containing a fragment subcloned from ZT1-1-A. Shown in the parentheses are the number of 54N1 transformants showing the *pwt2* phenotype (G+)/number of transformants tested. **d** Complementation test using F_1_ culture 54N1. Primary leaves of wheat cv. N4 were inoculated with Br48, 54N1 (showing the *PWT2* phenotype, G-), and 54N1 transformants carrying pACE1^Br48^ (54N1 + pACE1^Br48^) or empty vector (54N1 + EV), and incubated for 5 days at 22 °C. *N* = 26 (for 54N1 + pACE1^Br48^ #35) or 27 (for the other strains) biologically independent samples. **e**, **f** Disruption assay using wild isolate Br48. Primary leaves of wheat cv. N4 (**e**) and barley cv. H.E.S.4 (**f**) were inoculated with Br48, its *ACE1*-knockout mutants (Br48ΔACE1), and transformants of Br48ΔACE1(#112) carrying pACE1^Br48^ (Br48ΔACE1 + ACE1^Br48^), and incubated for 5 days at 22 °C. *N* = 17 (for Br48ΔACE1 #112 in (**f**)) or 18 (for the other strains in (**e** and **f**)) biologically independent samples. The boxplots in (**d**–**f**) show the percentage of lesion area in three independent experiments. Center lines show the medians; box limits indicate the 25th and 75th percentiles; whiskers extend to 1.5x the interquartile range from the 25th and 75th percentiles. Different letters indicate significant differences determined by Dunn’s test at the 5% level.

On primary leaves of wheat cv. Norin 4 (N4), PO12-7301-2 (*PWT1*; *PWT2*; *PWT5*) shows avirulence with the B- phenotype (discrete brown lesions) while Br48 (*pwt1*; *pwt2*; *pwt5*) shows virulence with the G+ phenotype (confluent green lesions)^23^. Out of the F_1_ cultures derived from PO12-7301-2 x Br48, we chose 54N1 (*pwt1*; *PWT2*; *pwt5*) showing the G- phenotype (discrete green lesions) peculiar to cultures with *PWT2* alone (virulent with weak aggressiveness) (Fig. 1d), backcrossed it with Br48 (virulent with strong aggressiveness), and produced 319 BC_1_F_1_ cultures (Fig. 1a). Molecular mapping suggested that the *Pwt2* locus was located on a region on chromosome 2 flanked by two markers, 613k_X and 488k_S (Fig. 1b). Sequence analyses in this region indicated that PO12-7301-2 did not carry additional genes compared with Br48, but lacked some genes harbored by Br48 (Fig. 1c). This result led us to an assumption that the functional allele at the *Pwt2* locus may not be *PWT2* derived from PO12-7301-2 but *pwt2* derived from Br48. To confirm this assumption, we screened a BAC library of Br48, selected two clones containing a part or most of this region, and introduced them into 54N1. Resulting transformants showed virulence with strong aggressiveness (G+) on N4 (Fig. 1c, Supplementary Fig. 1), indicating that *Pwt2* is a locus conditioning aggressiveness.

In the region covered by the shorter clone (ZT1-1-A), we found an allele of *ACE1*, which had been known to control avirulence of MoO isolates on a rice resistance gene, *Pi33*^25^. The *ACE1* allele in Br48 (*ACE1*^*Br48*^) had a complete ORF while its allele in PO12-7301-2 had a mutation leading to a stop codon (Fig. 1c). A fragment (~15 kb) containing *ACE1*^*Br48*^ was subcloned from ZT1-1-A to a plasmid vector, and the resulting plasmid (pACE1^Br48^) was introduced into 54N1. The transformants carrying pACE1^Br48^ gained the strong aggressiveness comparable to Br48 (Fig. 1c, d). Furthermore, a disruption of *ACE1*^*Br48*^ in Br48 impaired its aggressiveness, resulting in the G− phenotype, while a reintroduction of *ACE1*^*Br48*^ to the disruptant had it recover the strong aggressiveness (Fig. 1e and Supplementary Fig. 2). These results suggest that the core, functional gene at the *Pwt2* locus is *ACE1*^*Br48*^. GFSI1-7-2, another *PWT2* carrier, lacked most of the genes including *ACE1* in the candidate region (Fig. 1c).

Barley has been considered to be a common host of *P. oryzae* although its susceptibility to *P. oryzae* varies according to isolates and cultivars tested^30^. Barley cv. H.E.S.4 showed the G− and G+ phenotypes with 54N1 and Br48, respectively, as N4 did (Supplementary Table 1). In the BC_1_F_1_ population derived from 54N1 x Br48, G− and G+ cultures segregated in a 1:1 ratio on H.E.S.4, and the segregation pattern was perfectly concordant with that on N4 (Supplementary Table 1). Furthermore, the disruption of *ACE1*^*Br48*^ in Br48 and the reintroduction of *ACE1*^*Br48*^ to the disruptant resulted in the reduction and regain, respectively, of aggressiveness on H.E.S.4 as on N4 (Fig. 1f). Similar results were obtained not only at 22 °C (Fig. 1e, f) but also at 26 °C (Supplementary Fig. 3). These results suggest that *ACE1*^*Br48*^ is involved in the aggressiveness of MoT on both wheat and barley.

*ACE1* encodes a hybrid between a polyketide synthase and a non-ribosomal peptide synthetase (PKS-NRPS)^25^, and belongs to a cluster of 15 secondary metabolism genes (*ACE1* cluster)^26^ (Fig. 1c). The *ACE1* cluster is divided into Part A including *ACE1* and Part B including *SYN2* encoding another PKS-NRPS^31^ (Supplementary Fig. 4). Deletion of *SYN2*^*Br48*^ (an allele of *SYN2* in Br48) reduced its aggressiveness on N4 to an intermediate level, and reintroduction of *SYN2*^*Br48*^ to the deletion mutant had it regain the strong aggressiveness (Supplementary Fig. 4a, b). Furthermore, deletion of the whole cluster in Br48 followed by the reintroduction of its various portions suggested that the entire cluster is required for conferring the strong aggressiveness on N4 (Supplementary Figs. 4a, c, and 5). Taken together, we conclude that *Pwt2* is the *ACE1* cluster and is involved in the strong aggressiveness of Br48 (MoT). The *ACE1* cluster of Br48 (*Triticum* isolate) contained all of the genes designated in the *ACE1* cluster of Guy11 (*Oryza* isolate), and each of the Br48 genes was 99.5–100% identical to its corresponding Guy11 gene at the nucleotide and amino acid sequence levels (Supplementary Fig. 6).

### *ACE1*^*Br48*^ is involved in the suppression of the formation of fluorescent papilla

**Fig. 2:**
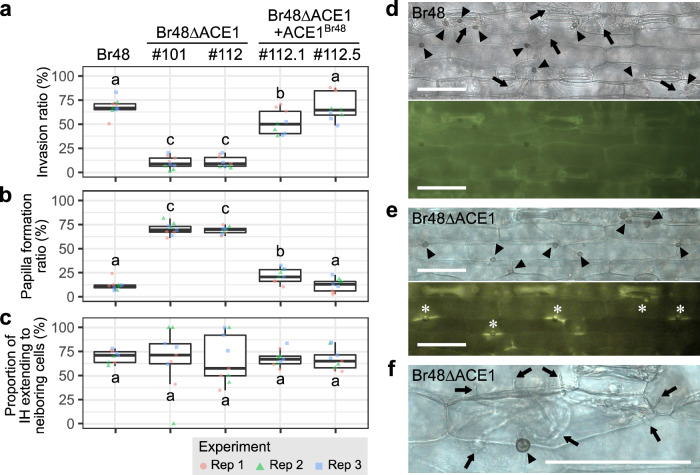
*ACE1*-knockout mutants are attenuated in penetration of wheat epidermal cell walls. Percentages of appressoria-producing infection hyphae (**a**), those inducing fluorescent papilla (**b**), and invasion hyphae extending to neighboring cells (**c**) in primary leaves of wheat cv. N4 inoculated with Br48, its *ACE1*-knockout mutants (Br48ΔACE1), and transformants of Br48ΔACE1(#112) carrying pACE1^Br48^ (Br48ΔACE1 + ACE1^Br48^), and incubated at 22 °C for 48 h. Data from three independent experiments (*n* = 9 biologically independent samples; three plants per experiment per strain) are shown in boxplots. Center lines show the medians; box limits indicate the 25th and 75th percentiles; whiskers extend to 1.5x the interquartile range from the 25th and 75th percentiles. Different letters indicate significant differences in mean ratios determined by the Tukey test at the 5% level. Representative responses of wheat epidermal cells to Br48 (**d**) and Br48ΔACE1#112 (**e**, **f**), 48 h after inoculation. Black arrowheads- appressoria; black arrows- infection hyphae; asterisks- fluorescent papilla. Bars indicate 100 µm.

To elucidate why the loss of function of *ACE1*^*Br48*^ leads to the G− phenotype, primary leaves of N4 were inoculated with Br48, its *ACE1*-knockout mutants (Br48ΔACE1), and transformants of Br48ΔACE1 carrying pACE1^Br48^ (Br48ΔACE1 + ACE1^Br48^). In leaves inoculated with Br48, more than 70% of germlings successfully invaded epidermal cells and extended infection hyphae (Fig. 2a, d). The incidence of fluorescent papillae was very low (Fig. 2b). By contrast, in those inoculated with Br48ΔACE1, more than 70% of germlings induced fluorescent papillae and failed to penetrate cell walls (Fig. 2b, e). However, a small proportion of germlings of Br48ΔACE1 evaded this reaction and penetrated cell walls (Fig. 2a). Once they entered the cells, they produced infection hyphae without inducing hypersensitive reactions, and extended the infection hyphae to the neighboring cells (Fig. 2c, f), which resulted in the peculiar phenotype, G− (discrete green lesions). In leaves inoculated with Br48ΔACE1 + ACE1^Br48^, the incidence of fluorescent papillae was suppressed, and many germlings successfully invaded epidermal cells (Fig. 2a, b). Taken together, cytological analyses with those disruptants and revertants suggested that the function of *ACE1*^*Br48*^ is associated with suppression of the formation of fluorescent papillae^22^ which inhibits cell wall penetration.

### Contribution of the *ACE1* cluster to aggressiveness of *P. oryzae* pathotypes is dependent on host species they attack

**Fig. 3:**
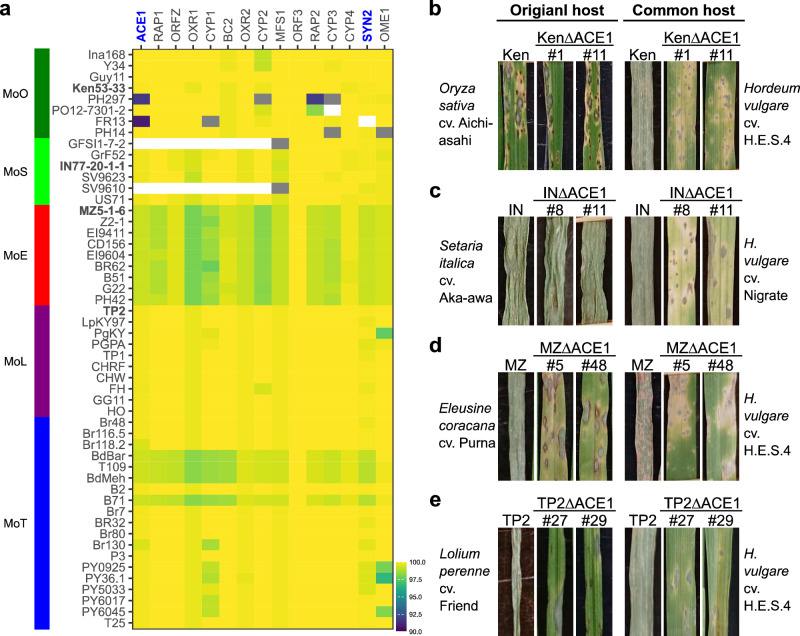
Conservation of the *ACE1* cluster in *P. oryzae* pathotypes correlates with its significance for their aggressiveness on host species. **a** Heatmap showing the identity of protein sequences of the *ACE1* cluster genes (x-axis) among *P. oryzae* isolates (y-axis). Their original hosts are indicated on the left with colored tiles. Shown in bold are representative isolates used in (**b**–**e**). **b** Pathogenicity of MoO isolate Ken53-33 (Ken) and its *ACE1*-knockout mutants (KenΔACE1) on rice (cv. Aichi–Asahi) and barley (cv. H.E.S.4). **c** Pathogenicity of MoS isolate IN77-20-1-1 (IN) and its *ACE1*-knockout mutants (INΔACE1) on foxtail millet (cv. Aka-awa) and barley (cv. Nigrate). Nigrate was used as a common host because IN77-20-1-1 was avirulent on H.E.S.4. **d** Pathogenicity of MoE isolate MZ5-1-6 (MZ) and its *ACE1*-knockout mutants (MZΔACE1) on finger millet (cv. Purna) and barley (cv. H.E.S.4). **e** Pathogenicity of MoL isolate TP2 and its *ACE1*-knockout mutants (TP2ΔACE1) on perennial ryegrass (cv. Friend) and barley (cv. H.E.S.4). In (**b**–**e**), inoculated seedlings were incubated at 26 °C (in rice) or 22 °C (the other plants) for 4–5 days. A compatible interaction leads to complete shriveling in barley, foxtail millet, finger millet, and perennial ryegrass, but does not in rice because fourth leaves were used in rice according to the standard protocol of rice infection assay with MoO.

Why could PO12-7301-2 (MoO) and GFSI1-7-2 (MoS), wild isolates lacking the functional *ACE1* or *ACE1* cluster, have survived in nature before isolation from natural fields? To answer this question, we first compared amino acid sequences of the *ACE1* cluster in wild isolates of various pathotypes (Fig. 3a). In MoT, MoL, and MoE all isolates carried *ACE1* alleles with highly conserved sequences. On the other hand, MoO and MoS contained some isolates that carried *ACE1* alleles with many mutations or lacked several cluster genes. We chose one isolate carrying an intact *ACE1* cluster from each pathotype (Ken53-33 from MoO, IN77-20-1-1 from MoS, MZ5-1-6 from MoE, and TP2 from MoL) and disrupted their *ACE1* alleles. On barley, the aggressiveness of the four wild isolates was all impaired by the disruption (Fig. 3b–e and Supplementary Fig. 7), suggesting that their *ACE1* alleles are functional and required for their aggressiveness on the common host. On their original hosts, however, the contribution of the *ACE1* alleles varied depending on host species. The aggressiveness of MZ5-1-6 (MoE) and TP2 (MoL) on finger millet and perennial ryegrass, respectively, was impaired by the disruption of their *ACE1* alleles (Fig. 3d, e and Supplementary Fig. 7). By contrast, the disruption of *ACE1* alleles of Ken53-33 (MoO) and IN77-20-1-1 (MoS) showed no effect on their aggressiveness on rice and foxtail millet, respectively (Fig. 3b, c, and Supplementary Fig. 7). These results suggest that the *ACE1* alleles are required for the infection of finger millet and perennial ryegrass, but not required for the infection of rice and foxtail millet. This is in accordance with a previous report that *ACE1* is not required for pathogenicity of MoO isolates on rice^25^. The results shown in Fig. 3b and c explain why MoO and MoS contained some isolates with mutated *ACE1* alleles or those lacking several cluster genes (Fig. 3a), and provide an answer to the question why PO12-7301-2 and GFSI1-7-2 could have survived in nature.

### *ACE1* alleles of MoT isolates are recognized by rice resistance gene *Pi33*

**Fig. 4:**
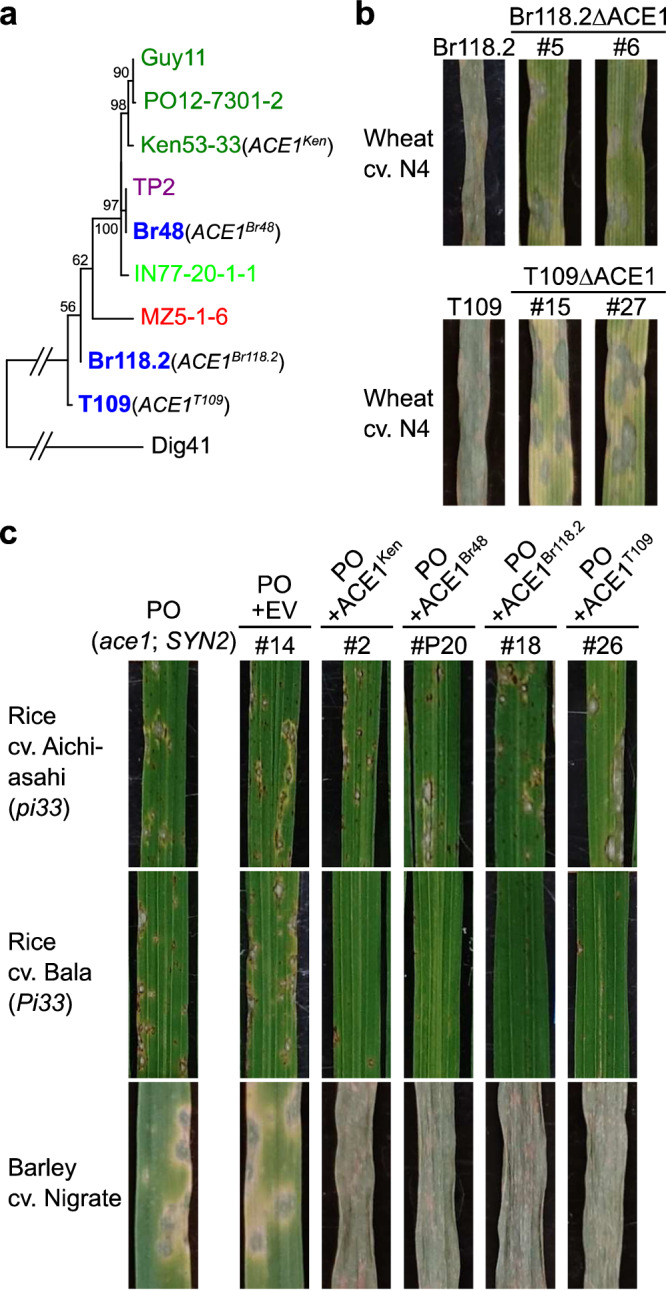
*ACE1* alleles of MoT isolates function as avirulence genes corresponding to rice resistance gene *Pi33.* **a** A maximum likelihood tree of *ACE1* alleles inferred from protein sequences. *ACE1* alleles are represented by names of isolates in which they were detected. Pathotypes of the isolates are color-coded as in Fig. 3a, i.e., MoT (blue), MoL (purple), MoE (red), MoS (light green), and MoO (green). Final designations of representative *ACE1* alleles are shown in parentheses. An *ACE1* homolog in *P. grisea* isolate Dig41 was used as an outgroup. **b** Effect of the disruption of *ACE1* alleles on aggressiveness of MoT isolates, Br118.2 and T109. Primary leaves of wheat cv. N4 were inoculated with Br118.2, T109, and their *ACE1*-knockout mutants (Br118.2ΔACE1 and T109ΔACE1), and incubated for 5 days at 22 °C. **c** Reactions of rice and barley to transformants of MoO isolate PO12-7301-2 (PO) carrying various *ACE1* alleles. Fourth leaves of rice cv. Aichi–Asahi (*pi33*) and cv. Bala (*Pi33*) and primary leaves of barley cv. Nigrate were inoculated with PO, its transformants carrying an empty vector (PO + EV), *ACE1* of MoO isolate Ken53-33 (PO + *ACE1*^*Ken*^), and the *ACE1* alleles of MoT isolates Br48, Br118.2, and T109 (PO + *ACE1*^*Br48*^, PO + *ACE1*^*Br118.2*^, and PO + *ACE1*^*T109*^), and incubated at 26 °C (rice) or 22 °C (barley) for 5 days. Similar results were obtained in three independent experiments.

*ACE1* is an avirulence gene corresponding to *Pi33* in the MoO-rice pathosystem^25^, and its alleles are widely distributed in the MoT population^32^. If these alleles in MoT are recognized by *Pi33*, it may be possible to use *Pi33* as a gene for resistance to wheat blast. We surveyed *ACE1* alleles of *Triticum* isolates in the databases and found two new types of alleles, *ACE1*^*Br118.2*^ and *ACE1*^*T109*^, in addition to *ACE1*^*Br48*^ (Fig. 4a and Supplementary Fig. 8). Disruption of *ACE1*^*Br118.2*^ and *ACE1*^*T109*^ impaired the aggressiveness of their carriers (Br118.2 and T109) on N4 (Fig. 4b and Supplementary Fig. 9), indicating that they are functional and required for the aggressiveness on wheat. To check interactions between these *ACE1* alleles and *Pi33*, we first chose PO12-7301-2 carrying the nonfunctional *ACE1* and a functional *SYN2* (Fig. 1c) as a recipient of those alleles. This isolate was virulent on both of rice cultivars, Aichi–Asahi (*pi33*) and Bala (*Pi33*) (Fig. 4c), but its transformants carrying *ACE1*^*Br48*^, *ACE1*^*Br118.2*^, and *ACE1*^*T109*^ gained complete avirulence on Bala without losing the virulence on Aichi–Asahi (Fig. 4c). These results suggest that all of these *ACE1* alleles function as avirulence genes corresponding to *Pi33*. On barley, those transformants gained strong aggressiveness as expected. Second, we chose PH297, an MoO isolate carrying nonfunctional *ACE1* and *SYN2* (Supplementary Fig. 10a), as another recipient. As test rice cultivars, CO39 (*pi33*) and IR64 (*Pi33*) were employed because PH297 was avirulent on Bala due to other factor(s). PH297 was virulent on both CO39 and IR64, but its transformants carrying *ACE1*^*Br48*^, *ACE1*^*Br118.2*^, and *ACE1*^*T109*^ again gained complete avirulence on IR64 in spite of lacking a functional *SYN2* (Supplementary Fig. 10b). These results suggest that the avirulence on *Pi33* is conferred by the *ACE1* alleles or the Part A alone, or in other words, that the *SYN2* allele is not required for the recognition by *Pi33*. This is in accordance with a previous report in the MoO - rice interaction that *SYN2* is not required for the avirulence on *Pi33* rice cultivars^26^. On barley those transformants did not gain strong aggressiveness, which is reasonable because *SYN2* is required for the fungal aggressiveness on wheat, a close relative of barley (Supplementary Fig. 4). Taken together, we suggest that, if *Pi33* is transferred from rice to wheat, it may recognize the *ACE1* alleles of MoT and function as a gene for resistance to MoT.

### A point mutation impairing the *ACE1* enzymatic function leads to reduced aggressiveness of MoT on wheat

**Fig. 5:**
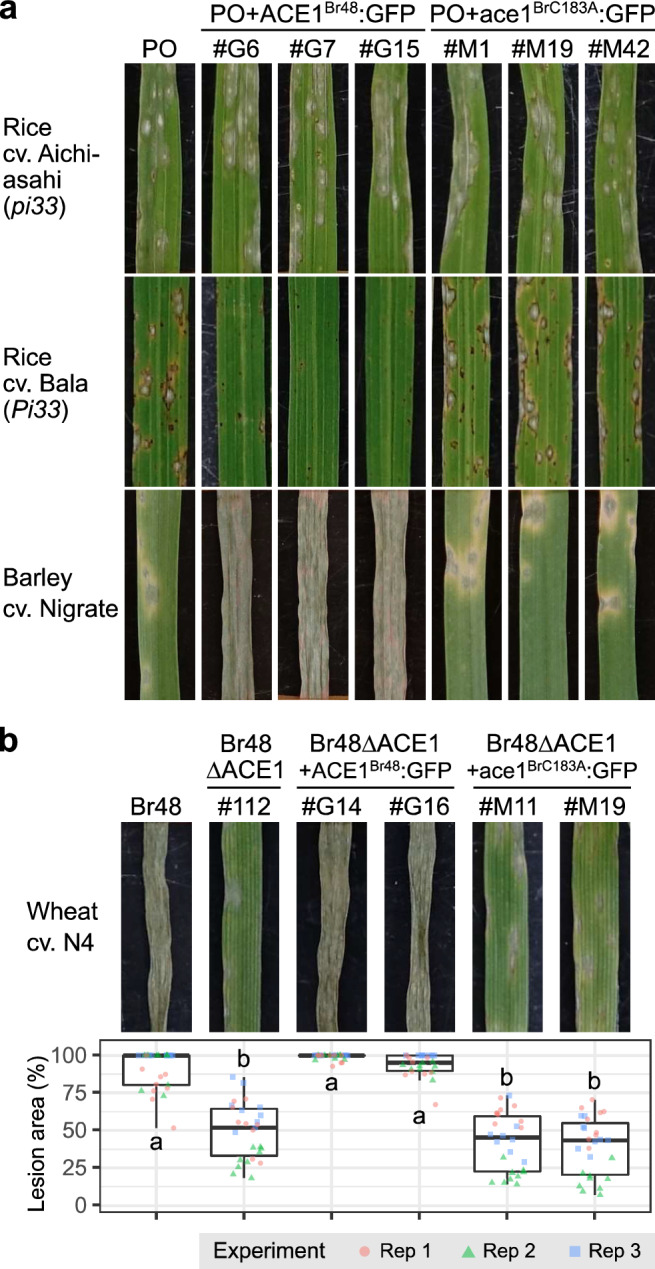
A point mutation in a core catalytic residue of *ACE1* abolishes not only the recognition by rice *Pi33* but also the aggressiveness on wheat. **a** Pathogenicity of MoO isolate PO12-7301-2 (PO), its transformants carrying intact *ACE1*^*Br48*^ fused to a GFP gene (PO + ACE1^Br48^:GFP), and those carrying *ACE1*^*Br48*^ with the C183A mutation fused to a GFP gene (PO + ace1^BrC183A^:GFP) on fourth leaves of rice cv. Aichi–Asahi (*pi33*) and cv. Bala (*Pi33*) and primary leaves of barley cv. Nigrate. Similar results were obtained in two independent experiments. **b** Pathogenicity of MoT isolate Br48, its *ACE1*-knockout mutant Br48ΔACE1 (#112), and transformants of Br48ΔACE1 (#112) carrying intact *ACE1*^*Br48*^ fused to a GFP gene (Br48ΔACE1 + ACE1^Br48^:GFP), and those carrying *ACE1*^*Br48*^ with the C183A mutation fused to a GFP gene (Br48ΔACE1 + ace1^BrC183A^:GFP) on primary leaves of wheat cv. N4. The boxplot shows the percentage of lesion area in three independent experiments. *N* = 26 (for Br48ΔACE1 + ACE1^Br48^:GFP #G16 and Br48ΔACE1+ace1^BrC183A^:GFP #M11) or 27 (for the other strains) biologically independent samples. Center lines show the medians; box limits indicate the 25th and 75th percentiles; whiskers extend to 1.5x the interquartile range from the 25th and 75th percentiles. Different letters indicate significant differences determined by Dunn’s test at the 5% level. Inoculated leaves were incubated at 26 °C (rice) or 22 °C (barley and wheat) for 5 days.

In the MoO-rice pathosystem, *Pi33* has been already defeated by some isolates^25,33^ probably through the loss of function of *ACE1* as exemplified by PO12-7301-2 and PH297 (Fig. 1c and Supplementary Fig. 10a). A critical concern about the use of *Pi33* in wheat breeding is that *Pi33* transferred to wheat may also be overcome by new races in future through mutations of the *ACE1* alleles in MoT. Böhnert et al.^25^ suggested that ACE1 enzymatic activity is essential for its avirulence function, based on a fact that a single amino acid exchange (C183A) in the β-ketoacyl synthase domain of ACE1 abolished the recognition of the fungus (MoO) by *Pi33*. Is the ACE1 enzymatic activity also essential for its function for aggressiveness? To answer this question, we introduced the C183A mutation into *ACE1*^*Br48*^, fused it to *GFP*, and established as *ace1*^*BrC183A*^:*GFP*. As a control, we also produced *ACE1*^*Br48*^:*GFP* by fusing the intact *ACE1*^*Br48*^ to GFP. These constructs were introduced into PO12-7301-2, and resulting transformants were sprayed on rice cultivars. On Bala (*Pi33*), transformants with *ACE1*^*Br48*^:GFP were avirulent but those with *ace1*^*BrC183A*^:*GFP* showed virulence with the same level of aggressiveness as PO12-7301-2 (Fig. 5a). On Aichi–Asahi (*pi33*) those transformants showed the same level of aggressiveness as PO12-7301-2 irrespective of the transgenes (Fig. 5a). On barley *ACE1*^*Br48*^:GFP conferred the strong aggressiveness on barley to MoO isolate PO12-7301-2, but *ace1*^*BrC183A*^:*GFP* did not, suggesting that the C183A mutation impaired the function for conferring strong aggressiveness on barley. In infection assay on wheat, Br48ΔACE1 was used as a recipient of those constructs. The expression of these constructs in appressoria was confirmed by fluorescence microscopy (Supplementary Fig. 11). *ACE1*^*Br48*^:*GFP* conferred the strong aggressiveness on wheat cv. N4 to Br48ΔACE1, but the C183A mutation impaired this function (Fig. 5b). These results suggest that *P. oryzae* can overcome *Pi33* through the single amino acid exchange without losing its fundamental aggressiveness on rice, but cannot do so on barley and wheat.

## Discussion

Böhnert et al.^25^ cloned *ACE1* as a gene conditioning the avirulence of MoO isolates on rice cultivars carrying *Pi33*. It was a unique avirulence gene encoding a hybrid enzyme involved in microbial secondary metabolism^25^. Collemare et al.^26^ found that *ACE1* is a member of a gene cluster composed of 15 secondary metabolism genes, and designated it as *ACE1* cluster. The *ACE1* cluster contained *SYN2* encoding another PKS-NRPS. These cluster genes displayed the same infection-specific expression pattern during the penetration of the fungus into host (rice) tissues^26^, suggesting that the gene cluster may play a role in the infection process. Based on the discontinuous distribution of the *ACE1* cluster among fungal species, Khaldi et al.^31^ speculated that the metabolite produced by this cluster may be an important pathogenicity factor. However, *ACE1* did not appear to contribute to lesion formation, invasive growth^25^, or aggressiveness on rice cultivars^26^. *SYN2* was not required for the biosynthesis of avirulence signal on *Pi33* nor aggressiveness on rice cultivars^26^. What is the intrinsic role of the *ACE1* cluster? The present study showed that the *ACE1* cluster plays an important role in aggressiveness of *P. oryzae* through promoting the successful penetration of cell walls of host plants (Fig. 2). The promotion of penetration was associated with the suppression of formation of fluorescent papillae. This is reasonable from the viewpoint of the timing of *ACE1* expression because *ACE1* transcription was detected only in mature appressoria, reaching to a maximum 17 h after inoculation^34^, and then rapidly disappeared once secondary infectious hyphae began to spread^25^. Interestingly, this contribution of *ACE1* to aggressiveness was apparent on barley, wheat, finger millet, and perennial ryegrass, but not detected on rice and foxtail millet (Fig. 3). This may be the reason why Böhnert et al.^25^ and Collemare et al.^26^ did not detect the contribution of *ACE1* or *SYN2* to aggressiveness or pathogenicity on rice. It should be noted that this difference of the contribution to aggressiveness is not attributable to inherent characteristics of the pathotypes or isolates but is dependent on plant species (genera) they attack. For example, the *ACE1* cluster in the MoO isolates does not contribute to their aggressiveness on rice (*Oryza sativa*), but contributes to their aggressiveness on barley (*Hordeum vulgare*) (Fig. 3). This may be attributable to difference of the sensitivity of each plant species (genus) to product(s) of the *ACE1* cluster. The product of the *ACE* cluster is supposed to be a tyrosine-derived cytochalasin compound^35^ but is not fully clarified. Further studies are needed to reveal how the product of the *ACE1* cluster promotes the successful penetration of cell walls or is involved in the suppression of papilla formation.

Most of newly introduced disease resistance genes in crops have been rendered ineffective by emergence of new pathogen races which escaped the recognition by the resistance genes through the loss of function or modification of their corresponding avirulence genes^36^. However, if an avirulence gene is indispensable for the survival of the pathogen and unable to accommodate itself so as to escape the recognition without losing its function for survival, the resistance gene should be durable^17,18^. We found that the *ACE1* cluster of MoT was indispensable for its strong aggressiveness on wheat, but also contained alleles of avirulence gene *ACE1* recognized by rice resistance gene *Pi33* (Fig. 4). Furthermore, a point mutation of the *ACE1* alleles in MoT for evading the recognition by *Pi33* inevitably caused critical reduction of its function for aggressiveness on wheat (Fig. 5). It seems impossible for MoT to evade the recognition by *Pi33* without losing its aggressiveness on wheat because both functions of *ACE1* (for avirulence and aggressiveness) are considered to be dependent on its enzymatic activity^25^ (Fig. 5). From these results, we suggest that *Pi33* may be durable if transferred from rice to wheat, or in other words, that *Pi33* may be used as an arrow targeting an Achilles heel of MoT.

Considering that resistance genes effective against MoT are rarely found in the wheat population, it is a promising strategy to look for such genes in rice which has a long history of interactions with MoO. Navia-Urrutia et al.^32^ found that a homolog of *AvrPiz-t* in MoT was recognized by *Piz-t* in rice. Then, they produced transgenic wheat plants expressing the rice *Piz-t* gene and inoculated them with a MoT isolate carrying the functional *AvrPiz-t* homolog. Although the transformants did not express a useful level of resistance in spikes, some of them showed a significant reduction of disease progress at the seedling stage. This result suggests that blast resistance genes in rice can be expressed and used in wheat. Since this strategy (transfer of rice genes to wheat) involves transgenic methods, the deficits such as low levels of expression or low levels of resistance in spikes will be overcome by manipulation of the transgene. *Pi33* has been mapped on the short arm of rice chromosome 8^33^ and narrowed down to its 240 kb region^37^, but not yet isolated. The cloning of *Pi33* is awaited for producing transgenic wheat plants carrying it and testing their reactions to wheat blast.

It should be noted that *Pi33* in rice has been already defeated by some MoO isolates^25,33^ as exemplified by PO12-7301-2 (Figs. 1, 4) and PH297 (Supplementary Fig. 10). This difference of predicted durability of *Pi33* in rice and wheat is attributable to the difference of contribution of *ACE1* alleles to aggressiveness on these crop species (Figs. 1e, 3b). The results shown in Fig. 3 further suggest that *Pi33* may also be used for breeding of barley, finger millet, and ryegrass as a durable resistance gene. Resistance genes that have been already defeated in one crop species may be revived as a durable resistance gene in other crop species.

## Methods

### Fungal materials and genetic crosses of *P. oryzae*

*Pyricularia oryzae* isolates used were PO12-7301-2 (MoO), Ken53-33 (MoO), PH297　(MoO), IN77-20-1-1 (MoS), MZ5-1-6 (MoE), TP2 (MoL), Br48 (MoT), Br118.2 (MoT), and T109 (MoT) (Supplementary Table 2). PH297 and T109 (original name, BTGP-6(e)) were provided by Dr. C. J. R. Cumagun, University of the Philippines, Los Banõs, the Philippines, and Dr. Md Tofazzal Islam, Bangabandhu Sheikh Mujibur Rahman Agricultural University, Bangladesh, respectively. In addition to these wild isolates, 54N1, an F_1_ culture derived from a cross between PO12-7301-2 and Br48^23^, was used to generate a BC_1_F_1_ population for linkage mapping of *Pwt2*. 54N1 was crossed with Br48 on oatmeal agar media as described previously^22^. One ascospore was isolated from each ascus so that each of the resulting hybrids was derived from an independent meiotic event.

### Plant materials

The plant materials used were *Triticum aestivum* cv. Norin 4 (N4), *Hordeum vulgare* cvs. H.E.S.4 and Nigrate, *Setaria italica* cv. Aka-awa (Si5), *Eleusine coracana* cv. Purna (Ec19), *Lolium perenne* cv. Friend, and *Oryza sativa* cvs. Aichi–Asahi, CO39, Bala, and IR64. Bala and IR64 were provided by the International Rice Research Institute (IRRI), the Philippines, and Dr. R. Ishikawa, Kobe University, Japan, respectively.

### Infection assay

Wheat and barley seeds were sown in vermiculite supplied with liquid fertilizer in a seedling case (5.5 × 15 × 10 cm), and grown at 22 °C in a controlled environment room with a 12 h photoperiod of fluorescent lighting for 8 days. Foxtail millet and finger millet were sown in Prime Mix soil (Sakata seed corporation, Yokohama, Japan) in the seedling case, and grown at 26 °C in a growth chamber with a 12 h photoperiod of fluorescent lighting for 2 weeks. Perennial ryegrass seeds were sown in Prime Mix soil in the seedling case, and grown at 22 °C in a phytotron chamber with a 12 h photoperiod of artificial lighting with metal halide lamps for 3 weeks. Rice seeds were sown in Ube soil (Zen-noh, Tokyo, Japan) in the seedling case, and grown at 26 °C in a natural-light cabinet for 3 weeks. Inocula were prepared as described previously^38^. Conidial suspension (1.0 × 10^5^ conidia per mL) with 0.01% Tween 20 was sprayed on primary (wheat and barley), third (foxtail millet and perennial ryegrass), and fourth (finger millet and rice) leaves fixed onto a hard plastic board with an air compressor. The cases were sealed to maintain high humidity and placed in darkness for 24 h at 22 °C or 26 °C. The inoculated seedlings were then transferred to a growth chamber with a 12 h photoperiod of fluorescent lighting and incubated further at the same temperature as in darkness. Four to five days after inoculation, symptoms were evaluated based on the lesion areas formed by fungal infection. For each leaf, the leaf size and lesion area were measured using ImageJ (https://imagej.nih.gov/ij/), and the relative area covered by lesions (percentage of lesion area) was calculated.

### Construction of a BAC library

Genomic DNA of Br48 was digested with Sau3AI, and ~100 kb fragments were ligated with pCC1BAC/BamHI vector (EPICENTRE). The ligation products were transformed into *E. coli* ElectroMAX DH10B (Invitrogen) through electroporation.

### Construction of plasmids

Details of the plasmid construction and the primer sequences used are described in Supplementary Table 3. Their outlines are briefly described below. To construct an *ACE1*^*Br48*^ complementation vector, a ~ 15 kb EcoRI fragment containing ~1.6 kb upstream, ORF, and ~1.4 kb downstream sequences of *ACE1*^*Br48*^ was liberated from ZT1-1-A (a BAC clone of the Br48 genomic library), cloned into pBlueScript II SK(+) and established as pACE1^Br48^. Other *ACE1*/*SYN2* complementation vectors were constructed by assembling genomic fragment(s) containing ~1.6–1.7 kb upstream, ORF, and ~1.4 kb downstream sequences and a vector backbone of pBlueScript II SK(+) via In-Fusion cloning (Takara Bio, Otsu, Japan). These genomic fragments were either PCR-amplified from genomic DNA of *P. oryzae* isolates or liberated from a BAC clone of Br48.

To generate *ACE1*-KO (knock out) mutants, a KO vector was constructed by replacing a ~ 3.6-kb AgeI fragment of pACE1^Br48^ with a hygromycin resistance gene cassette. The hygromycin resistance gene cassette was PCR-amplified from pSH75^39^ with primers HygR_F2 and HygR_R2. To generate *SYN2*- and *ACE1* cluster-KO mutants, KO vectors were constructed by assembling ~2.5-3.1-kb upstream and downstream fragments of the target gene/region, a hygromycin resistance gene cassette, and a vector backbone of pBlueScript II SK(+) via In-Fusion cloning. To construct CRISPR/Cas9 expression vectors targeting genes/regions of interest, sense, and antisense oligonucleotides were annealed and inserted to pCRISPR/Cas-U6-1^40^ by Golden Gate cloning as described in Arazoe et al.^40^.

To monitor the expression of ACE1 proteins, we constructed a vector encoding a C-terminal fusion of ACE1 with GFP and expressed under the control of the ACE1 promoter and terminator sequences. A 16.4 kb NotI fragment containing ~1.6 kb upstream and N-terminal part of the coding sequences of *ACE1*^*Br48*^ and the vector backbone of pBlueScript II SK(+) was liberated from pACE1^Br48^. The C-terminal part of the *ACE1*^*Br48*^ coding sequence lacking the stop codon was PCR-amplified from pACE1^Br48^ with primers pACE1_N_F and ACE1woStop_R. A ~ 0.7 kb *GFP* gene fragment was PCR-amplified from pEGFP75^41^ with primers GFP_i_F and GFP_i_R. A ~ 1.4-kb downstream sequence of *ACE1*^*Br48*^ was PCR-amplified from pACE1^Br48^ with primers ACE1ter_F and pACE1_N_R. The four fragments were assembled by InFusion reaction to establish pACE1^Br48^-GFP.

To introduce C183A mutation in ACE1^Br48^-GFP, a DNA fragment carrying “TGC” to “GCT” mutation in the codon position for the 183rd amino acid of ACE1^Br48^ was generated by annealing two oligos, F_C183A and R_C183A. Upstream and downstream flanking sequences of the mutation target site were PCR-amplified from pACE1^Br48^-GFP with primers ACE1_m_F1 and ACE1_m_R1 and ACE1_m_F2 and ACE1_m_R2, respectively. To replace the corresponding sequence in pACE1^Br48^-GFP, the three DNA fragments were integrated via InFusion reaction with an 18.6 kb BspEI/FseI double-digestion fragment liberated from pACE1^Br48^-GFP. The resultant plasmid was established as pACE1^BrC183A^-GFP.

### Transformation of *P. oryzae*

Protoplasts of *P. oryzae* were prepared and transformed as described previously^42^. In the complementation tests, vectors carrying genes from the *ACE1* cluster were introduced into *P. oryzae* isolates/strains through co-transformation with pSH75 containing the *hph* gene for hygromycin-B selection or pII99^43^ containing the *nptII* gene for geneticin selection. In targeted gene disruption, KO vectors were introduced alone or together with CRISPR/Cas9 expression vectors into protoplasts. Transformants carrying transgenes were selected by colony PCR. Details of the transformants produced in this study are described in Supplementary Table 4.

### Microscopy

For microscopic observation of infection behaviors of *ACE1*-KO mutants, primary leaves of wheat cv. N4 were sampled at 48 h after inoculation and boiled in alcoholic lactophenol (lactic acid/phenol/glycerol/distilled water/ethanol = 1:1:1:1:8, vol/vol/vol/vol/vol) for 2 min for fixation. Cytological response of the specimens was observed under bright and dark fields of a fluorescence microscope (Olympus, Tokyo, Japan) with an exciter filter B. Fluorescence of the ACE1-GFP fusion protein was observed using the fluorescence microscope at 20 h after inoculation of glass coverslips with conidial suspensions.

### Detection of *ACE1* cluster genes and phylogenetic analysis

Protein sequences of 15 genes within the *ACE1* cluster (Fig. 3) were retrieved from the genome annotation of *P. oryzae* strain 70-15 (version MG8). The protein sequences were aligned to genome assemblies of *P. oryzae* isolates listed in Supplementary Tables 2 and 5 via exonerate^44^ with “-m p2g --percent 50 -n 1” options. Homologous sequences in each isolate and the identity to the query protein sequence were retrieved from the exonerate output. Protein sequences of the *ACE1* alleles from different isolates were aligned by MUSCLE^45^ in MEGA version 11^46^. A maximum likelihood tree was inferred by IQtree version 2.2.0.3^47^ and branch supports were obtained with the ultrafast bootstrap^48^. The phylogenetic tree was visualized by using iTOL^49^.

### Statistics and reproducibility

For the assessment of fungal virulence, data from 17-45 biologically independent samples from three independent experiments were collected per strain, except for Supplementary Figs. 7b (13-16 biologically independent samples from two independent experiments per strain) and 9a (9-17 biologically independent samples from a single experiment per strain). For the assessment of the host invasion behavior, data from 9 biologically independent samples from three independent experiments were collected per strain. Significant differences between groups were determined using the Dunn’s test (Figs. 1d, e, f, and 5b, and Supplementary Figs. 3a, b, 4b, c, 7a, b, c, d, 9a, and b) or Tukey test (Fig. 2a, b, and c) at 5% level.

### Reporting summary

Further information on research design is available in the Nature Portfolio Reporting Summary linked to this article.

### Supplementary information

Supplementary information

Description of Additional Supplementary Files

Supplementary Data 1

Reporting Summary

## Data Availability

Nucleotide sequence data reported are available in the DDBJ Sequenced Read Archive under the accession number PRJDB14584. Source data and original gel images are available in Supplementary Data 1 and Supplementary Fig. 12, respectively. Any additional data supporting the findings of this study are available from the corresponding author upon reasonable request. Fungal genome sequence data used in this study are listed in Supplementary Table 5. All plasmids, plant lines, and fungal strains employed or generated in this work are available from the authors upon request.
