# Supplementary material for: The ACE1 secondary metabolite gene cluster is a pathogenicity factor of wheat blast fungus
Source: Commun Biol. 2024 Jul 4;7:812. doi: 10.1038/s42003-024-06517-7 (PMC11224330; doi:10.1038/s42003-024-06517-7)
Supplement: Supplementary file 1 — Supplementary information [file 42003_2024_6517_MOESM1_ESM.pdf]

The *ACE1* secondary metabolite gene cluster is a pathogenicity factor of wheat blast fungus

Trinh T. P. Vy, Yoshihiro Inoue, Soichiro Asuke, Izumi Chuma, Hitoshi Nakayashiki, Yukio Tosa

This PDF file includes:

Supplementary Figures 1 to 12

Supplementary Tables 1 to 5

References cited in Supplementary Figures and Tables

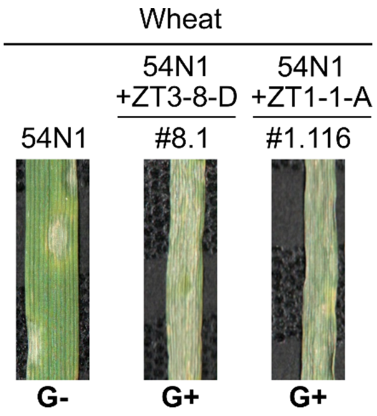

Supplementary Fig. 1. Introduction of Br48 BAC clones containing the *Pwt2* locus confers strong aggressiveness on wheat. Primary leaves of wheat cv. N4 were inoculated with 54N1 (showing the *PWT2* phenotype, G-) and 54N1 transformants carrying Br48 BAC clones (54N1+ZT3-8-D and 54N1+ZT1-1-A), and incubated at 22°C for 5 days.

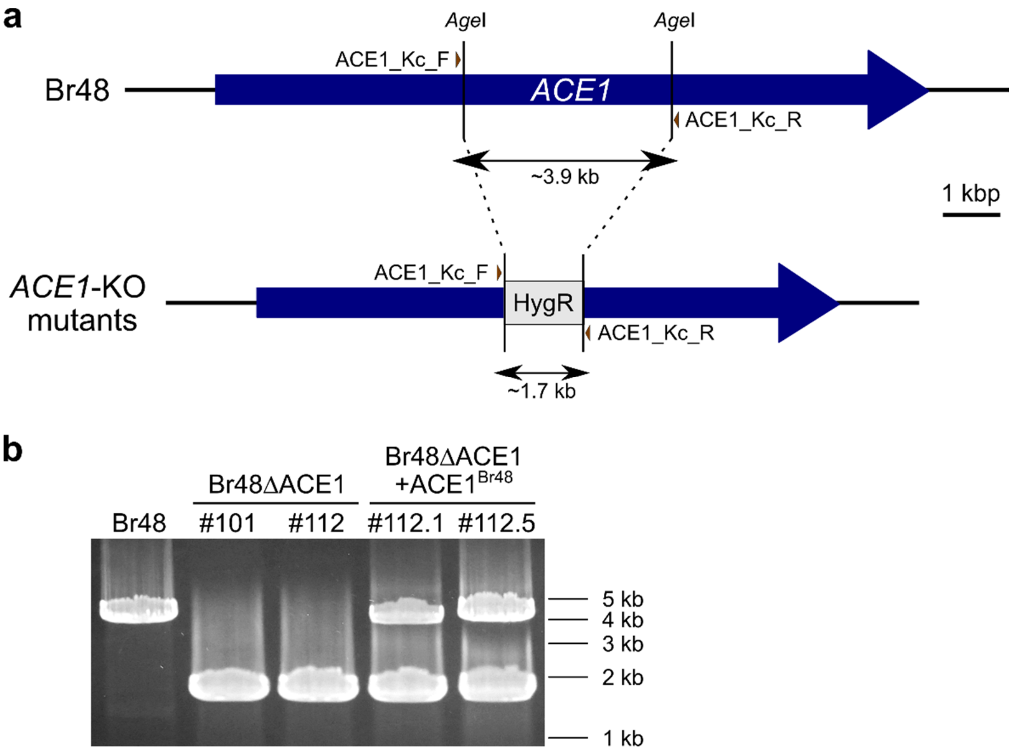

Supplementary Fig. 2. Generation of *ACE1* knockout mutants of Br48 and reintroduction of *ACE1*^{Br48} (intact *ACE1* derived from Br48) to the mutants. a Schematic representation of the *ACE1* locus in Br48 and its *ACE1*-knockout mutants. Arrowheads indicate primers used in (**b**). **b** Verification of *ACE1*-knockout and reintroduction of *ACE1*^{Br48} by colony PCR. Genomic DNA was used as a template for PCR amplification with primers ACE1_Kc_F (5’-CGAAATACCCCGACAGCATC-3’) and ACE1_Kc_R (5’-ATGCAGAAGTCGTGGGAGAG-3’), and PCR amplicons were run on a 0.7 % agarose gel.

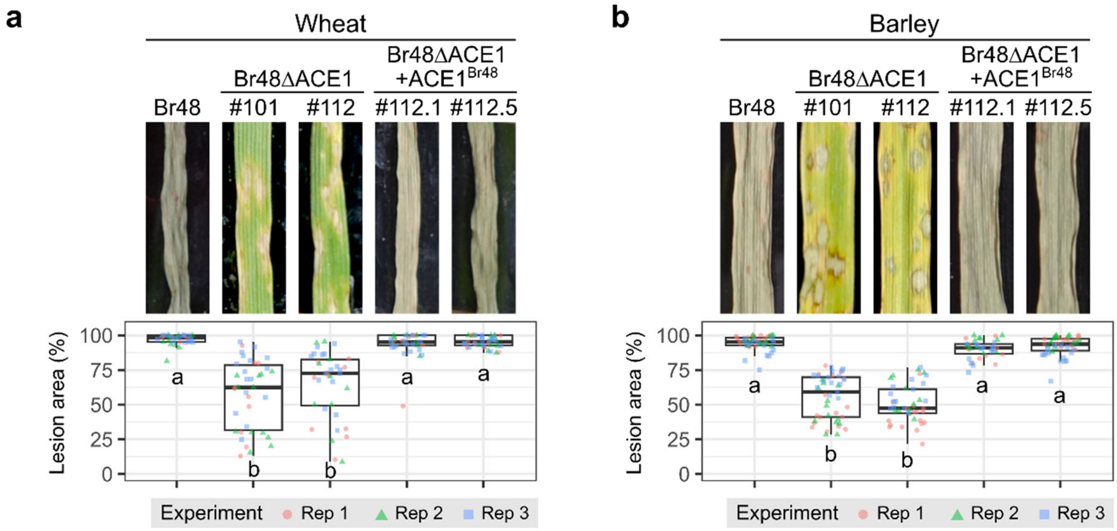

Supplementary Fig. 3. *ACE1* is required for conferring strong aggressiveness on wheat and barley even at a high temperature condition. Primary leaves of wheat cv. N4 (a) and barley cv. H.E.S.4 (b) were inoculated with Br48, its ACE1-knockout mutants (Br48ΔACE1), and transformants of Br48ΔACE1(#112) carrying pACE1^{Br48} (Br48ΔACE1+ACE1^{Br48}), and incubated for 5 days at 26 °C. The boxplots show the percentage of lesion area in three independent experiments (n = 32, 38, 36, 34, and 38 (**a**) and 45, 45, 43, 37, and 43 (**b**) biologically independent samples). Center lines show the medians; box limits indicate the 25th and 75th percentiles; whiskers extend to 1.5x the interquartile range from the 25th and 75th percentiles. Different letters indicate significant differences determined by Dunn's test at the 5 % level.

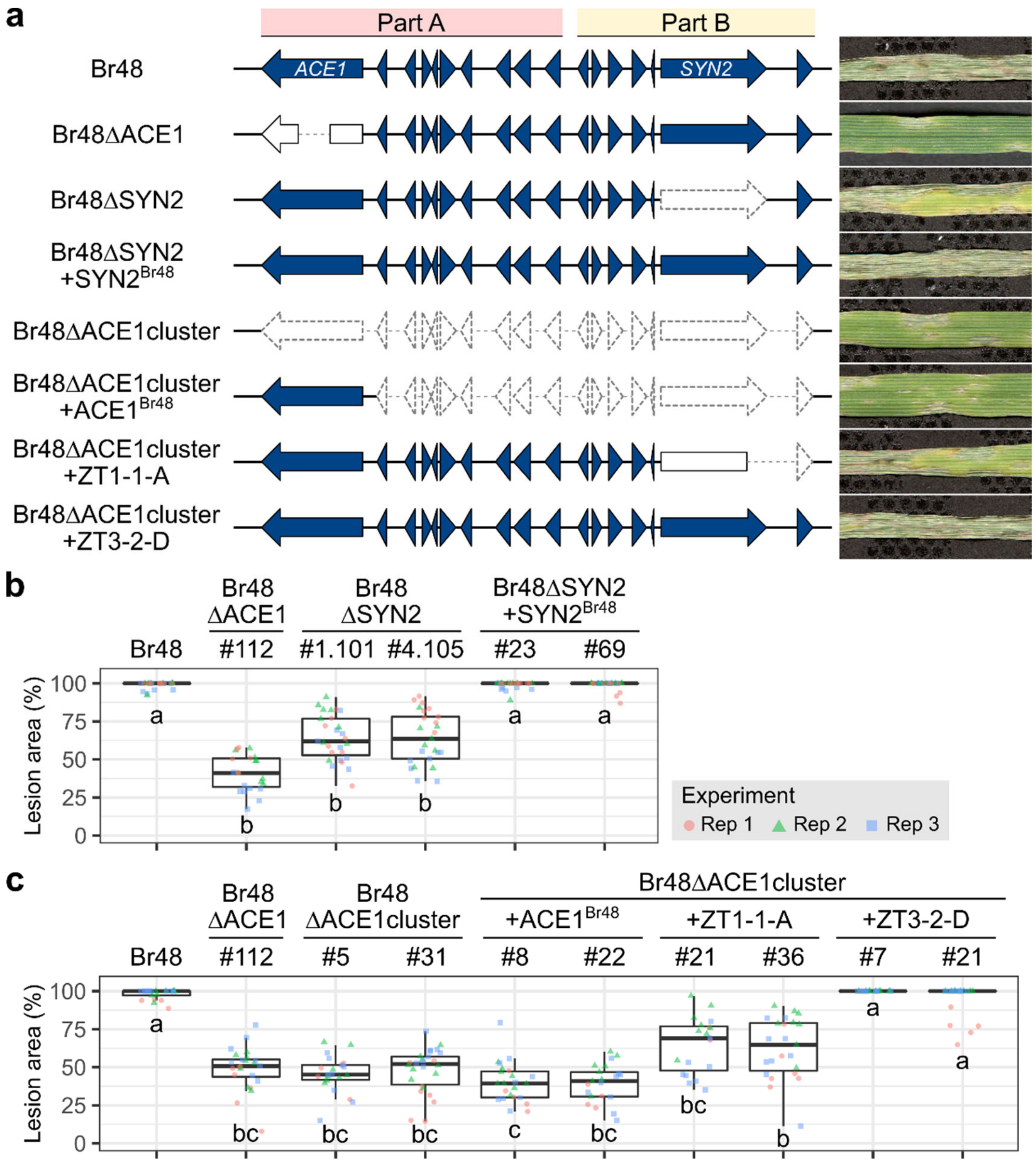

Supplementary Fig. 4. The entire *ACE1* cluster is required for conferring full virulence on wheat. a Schematic representation of partial/complete knockout mutants of Br48 and their aggressiveness. The arrows and arrowheads painted in blue are intact genes while those drawn with dotted lines are deleted genes. ZT3-2-D is a Br48 BAC clone containing the entire cluster. These mutants were sprayed on primary leaves of wheat cv. N4 and incubated at 22 °C for 5 days. **b** Percentage of lesion area formed by Br48, its *ACE1*-knockout mutant (Br48ΔACE1), *SYN2*-knockout mutants (Br48ΔSYN2), and transformants of Br48ΔSYN2 (#4.105) carrying *SYN2*^{Br48} (Br48ΔSYN2+SYN2^{Br48}). The boxplot shows the percentage of lesion area in three independent experiments (n = 25, 23, 27, 25, 26, and 27 biologically independent samples). Center lines show the medians; box limits indicate the 25th and 75th percentiles; whiskers extend

to 1.5x the interquartile range from the 25th and 75th percentiles. Different letters indicate significant differences determined by Dunn's test at the 5% level. **c** Percentage of lesion area formed by Br48, its *ACE1*-knockout mutant (Br48ΔACE1), *ACE1* cluster-knockout mutants (Br48ΔACE1cluster), transformants of Br48ΔACE1cluster (#31) carrying $ACE1^{Br48}$ (Br48ΔACE1cluster+ $ACE1^{Br48}$), those carrying a Br48 BAC clone (ZT1-1-A) with a partial *ACE1* cluster (Br48ΔACE1cluster+ZT1-1-A), and those carrying a Br48 BAC clone (ZT3-2-D) with the entire *ACE1* cluster (Br48ΔACE1cluster+ZT3-2-D). The boxplot shows the percentage of lesion area in three independent experiments (n = 21, 24, 22, 26, 23, 21, 19, 23, 20, and 22 biologically independent samples). Different letters indicate significant differences determined by Dunn's test at the 5% level.

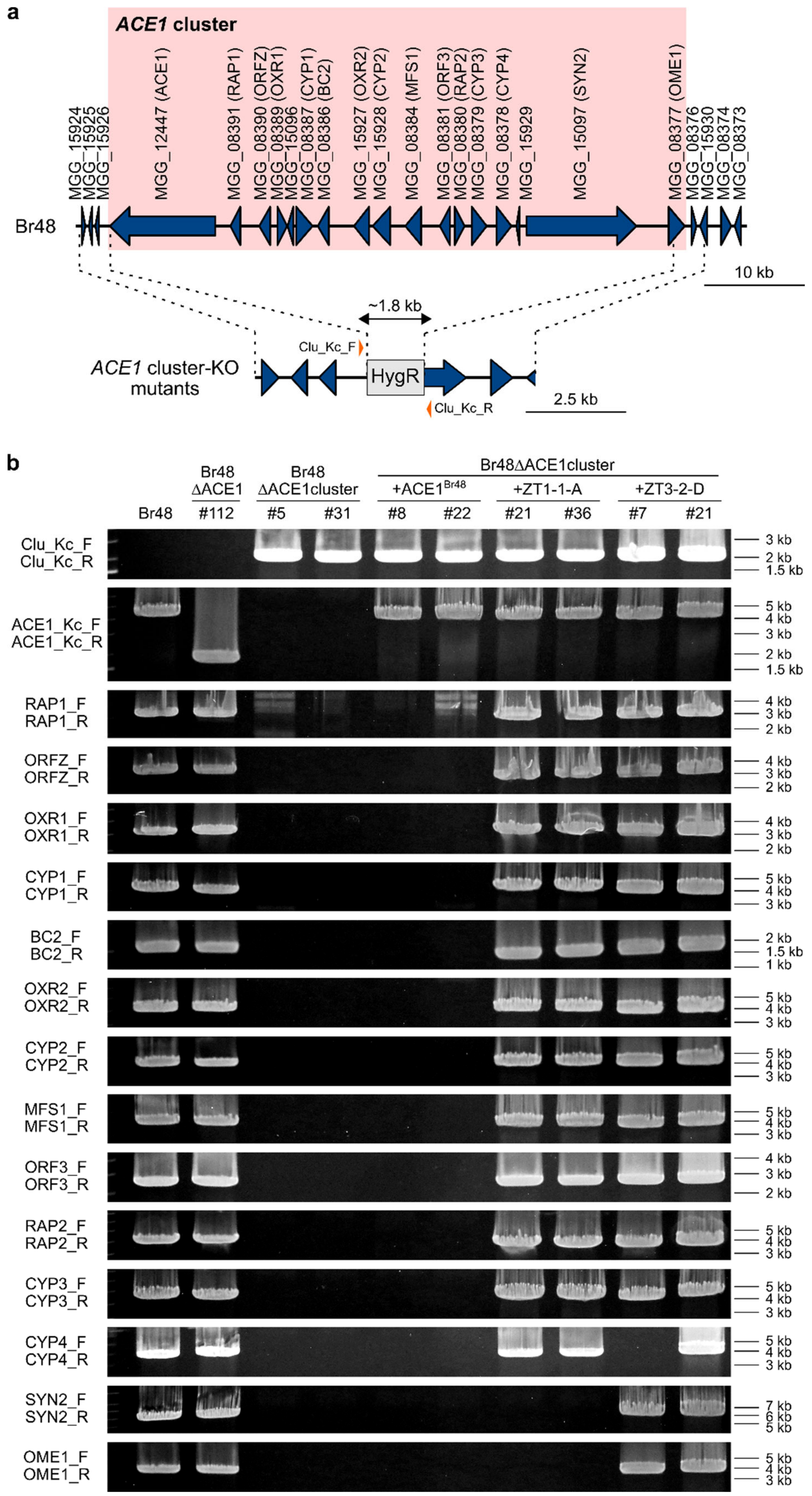
a
ACE1 cluster
MGG_15924
MGG_15925
MGG_15926
MGG_12447 (ACE1)
MGG_08391 (RAP1)
MGG_08390 (ORFZ)
MGG_08389 (OXR1)
MGG_15096
MGG_08387 (CYP1)
MGG_08386 (BC2)
MGG_15927 (OXR2)
MGG_15928 (CYP2)
MGG_08384 (MFS1)
MGG_08381 (ORF3)
MGG_08380 (RAP2)
MGG_08379 (CYP3)
MGG_08378 (CYP4)
MGG_15929
MGG_15097 (SYN2)
MGG_08377 (OME1)
MGG_08376
MGG_15930
MGG_08374
MGG_08373
Br48
10 kb
~1.8 kb
Clu_Kc_F
ACE1 cluster-KO mutants
HygR
Clu_Kc_R
2.5 kb
b
Br48
Br48 ΔACE1 #112
Br48 ΔACE1cluster #5 #31
Br48ΔACE1cluster
+ACE1Br48 #8 #22
+ZT1-1-A #21 #36
+ZT3-2-D #7 #21
Clu_Kc_F Clu_Kc_R
ACE1_Kc_F ACE1_Kc_R
RAP1_F RAP1_R
ORFZ_F ORFZ_R
OXR1_F OXR1_R
CYP1_F CYP1_R
BC2_F BC2_R
OXR2_F OXR2_R
CYP2_F CYP2_R
MFS1_F MFS1_R
ORF3_F ORF3_R
RAP2_F RAP2_R
CYP3_F CYP3_R
CYP4_F CYP4_R
SYN2_F SYN2_R
OME1_F OME1_R
1 kb
1.5 kb
2 kb
3 kb
4 kb
5 kb
6 kb
7 kb

Supplementary Fig. 5. Generation of *ACE1* cluster knockout mutants of Br48 and reintroduction of the (partial) *ACE1* cluster. a Schematic representation of the *ACE1* cluster in Br48 and its knockout mutants. Orange arrowheads indicate primers used in **b**. **b** Verification of *ACE1* cluster knockout and reintroduction of 15 genes in the *ACE1* cluster by colony PCR. Genomic DNA was used as a template for PCR amplification with primers listed below, and PCR amplicons were run on a 0.7 % agarose gel. For verification of the *ACE1* cluster knockout, primers Clu_Kc_F (5'-CATGGCCGTGTGAGCATATG-3') and Clu_Kc_R (5'-ATGGTGCGTTGGCTGGTTTTG-3') were used for PCR. To check the introduction of genes in the *ACE1* cluster, following primer pairs were used: ACE1_Kc_F and ACE1_Kc_R for *ACE1*, RAP1_F (5'-GGAAGTACTAGGACCCTGGC-3') and RAP1_R (5'-AATCAAAATTGCCCGCCTGT-3') for *RAP1*, ORFZ_F (5'-GGCGCTGACAAAATCCTTGA-3') and ORFZ _R (5'-TTGAACAAAGGCCACGAGAC -3') for *ORFZ*, OXR1_F (5'- ATCTCCCTCGACCTTGGATC-3') and OXR1_R (5'- TGGCCTGTTATTTTCCGCAG-3') for *OXR1*, CYP1_F (5'-AACAGATTCGGTGAGCTTGC-3') and CYP1_R (5'- TCACCGGCTGAGCTACAAAT-3') for *CYP1*, BC2_F (5'- CGAACGTGAGCCAAGCATTA-3') and BC2_R (5'-GTCGCAATTACGCAAAAGCC-3') for *BC2*, OXR2_F (5'- CAGCTACCAAAACCGACGAG-3') and OXR2_R (5'- GGAGGGACACTTTGGGGTTA-3') for *OXR2*, CYP2_F (5'-TTCGTGGCTTTGCTTGTACC-3') and CYP2_R (5'- GGTGCAAAAGTTCAGGCTGA-3') for *CYP2*, MFS1_F (5'- GAGCCGCCGAATATTGTTGA-3') and MFS1_R (5'-TCCCGATGCTGAAAGGTAGG-3') for *MFS1*, ORF3_F (5'- ACGTCAGTGAAAGGGTCGAT-3') and ORF3_R (5'- TCTGATTCGGCTCCCAAAGT-3') for *ORF3*, RAP2_F (5'-TCTGATTCGGCTCCCAAAGT-3') and RAP2_R (5'- TGAAACGGAGCAAAAGTCGG-3') for *RAP2*, CYP3_F (5'- ATCGACCCTTTCACTGACGT-3') and CYP3_R (5'-TACCCAGCCACCTACCTAGT-3') for *CYP3*, CYP4_F (5'- GCCTACAGACACCTTCACCT-3') and CYP4_R (5'- TGCATCACATCTTCGTCACG-3') for *CYP4*, SYN2_F (5'-GAGGCTTTGCAGGTCGAAAA-3') and SYN2_R (5'- TCTCCCTTTTGCAACATGGC-3') for *SYN2*, and OME1_F (5'- GTGGGTGGTCTGGCTAATT-3') and OME1_R (5'-CGTGTCAAAGCAACAACCA-3') for *OME1*. Reintroduction of *CYP4* could not be verified in the strain Br48ΔACE1cluster+ZT3-2-D (#7), indicating that this strain carries the *ACE1* cluster lacking *CYP4*.

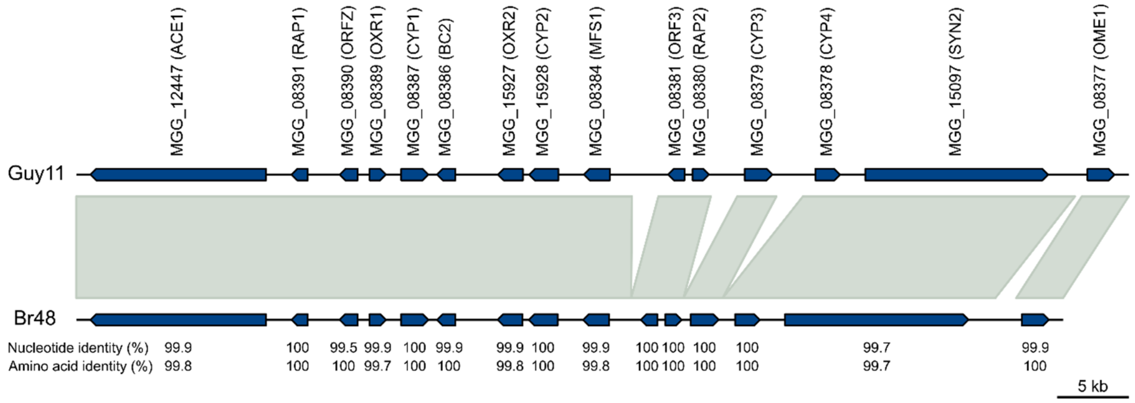

Supplementary Fig. 6. Comparison of the *ACE1* cluster between *Oryza* isolate Guy11 and *Triticum* isolate Br48. Syntenic regions are indicated by gray-shaded regions. Directional boxes indicate genes in the respective orientation. Numbers below each gene indicate percentages of identity at the nucleotide (coding sequence) and amino acid levels.

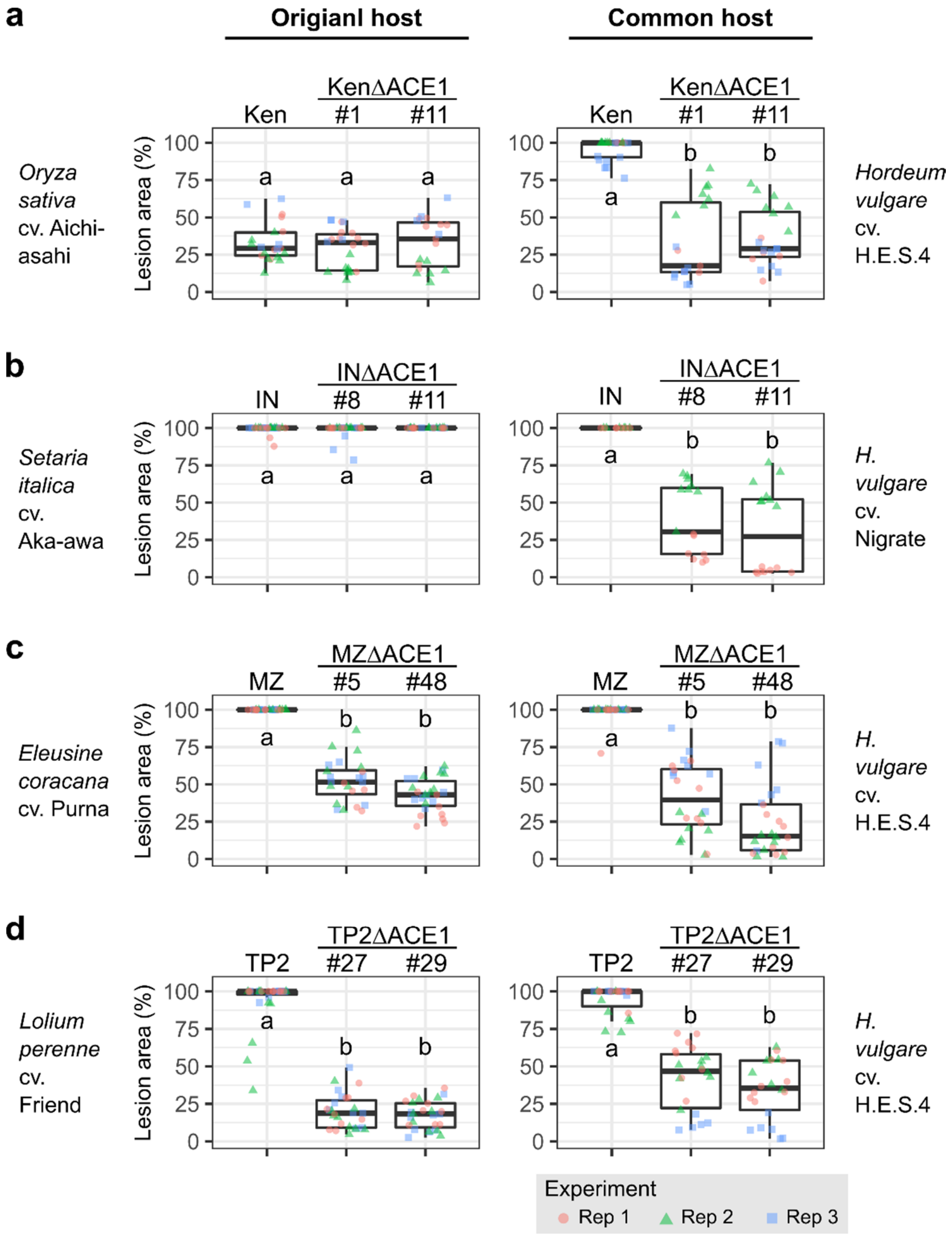

Supplementary Fig. 7. Quantitative evaluation of the effect of the disruption of *ACE1* alleles on aggressiveness of *P. oryzae* pathotypes on different host species. a Percentages of lesion area formed by MoO isolate Ken53-33 (Ken) and its *ACE1*-knockout mutants (KenΔACE1) on rice (cv. Aichi-asahi; n = 21, 22, and 19 biologically independent samples) and barley (cv. H.E.S.4; n = 21, 19, and 20 biologically independent samples). **b** Percentages of lesion area formed by MoS isolate IN77-20-1-1 (IN) and its *ACE1*-knockout mutants (INΔACE1) on foxtail millet (cv. Aka-awa; n = 27 biologically independent samples) and barley (cv. Nigrate; n = 13, 15, and 16 biologically independent samples). **c** Percentages of lesion area formed by MoE

isolate MZ5-1-6 (MZ) and its *ACE1*-knockout mutants (MZΔACE1) on finger millet (cv. Purna; n = 27, 24, and 26 biologically independent samples) and barley (cv. H.E.S.4; n = 25, 24, and 24 biologically independent samples). **d** Percentages of lesion area formed by MoL isolate TP2 and its *ACE1*-knockout mutants (TP2ΔACE1) on perennial ryegrass (cv. Friend; n = 27 biologically independent samples) and barley (cv. H.E.S.4; n = 23, 22, and 22 biologically independent samples). The boxplots show the percentage of lesion area in two or three independent experiments (seven to nine plants per experiment per strain). Center lines show the medians; box limits indicate the 25th and 75th percentiles; whiskers extend to 1.5x the interquartile range from the 25th and 75th percentiles. Different letters indicate significant differences determined by Dunn's test at the 5 % level.

a

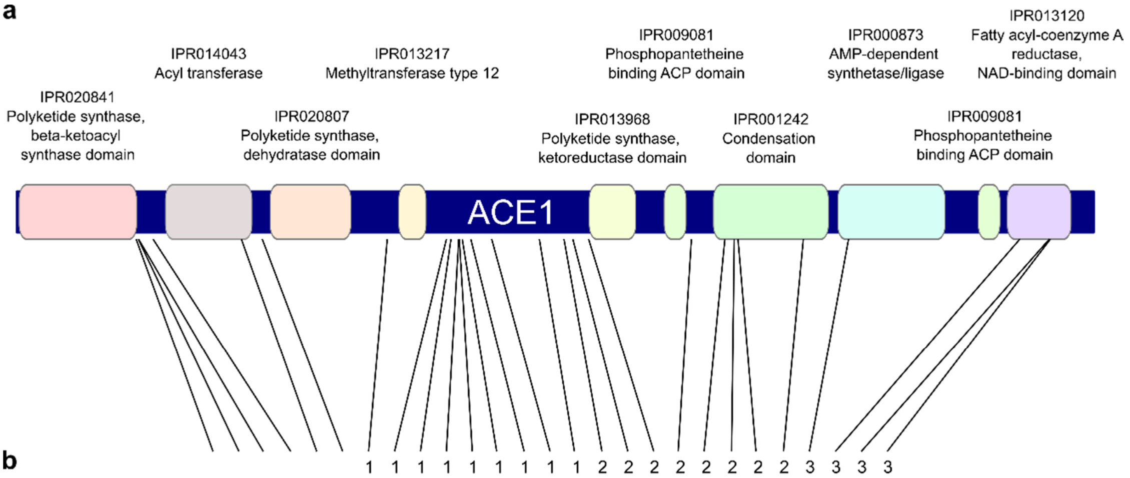

b

Isolate	450	458	459	513	843	921	1388	1609	1626	1655	1657	1668	1708	1778	1956	2049	2081	2140	2524	2649	2684	2698	2942	3111	3751	3861	3865	ACE1 type
Br48	T	V	T	V	S	G	A	H	T	S	A	H	F	H	Q	C	D	R	Q	G	A	F	H	T	G	A	G	ACE1^{Br48}
Br116.5	.	.	.	.	.	.	.	.	.	.	.	.	.	.	.	.	.	.	.	.	.	.	.	.	.	.	.	ACE1^{Br48}
B2	.	.	.	.	.	.	.	.	.	.	.	.	.	.	.	.	.	.	.	.	.	.	.	.	.	.	.	ACE1^{Br48}
BR32	.	.	.	.	.	.	.	.	.	.	.	.	.	.	.	.	.	.	.	.	.	.	.	.	.	.	.	ACE1^{Br48}
Br7	.	.	.	.	.	.	.	.	.	.	.	.	.	.	.	.	.	.	.	.	.	.	.	.	.	.	.	ACE1^{Br48}
Br80	.	.	.	.	.	.	.	.	.	.	.	.	.	.	.	.	.	.	.	.	.	.	.	.	.	.	.	ACE1^{Br48}
P3	.	.	.	.	.	.	.	.	.	.	.	.	.	.	.	.	.	.	.	.	.	.	.	.	.	.	.	ACE1^{Br48}
PY0925	.	.	.	.	.	.	.	.	.	.	.	.	.	.	.	.	.	.	.	.	.	.	.	.	.	.	.	ACE1^{Br48}
PY36.1	.	.	.	.	.	.	.	.	.	.	.	.	.	.	.	.	.	.	.	.	.	.	.	.	.	.	.	ACE1^{Br48}
PY5033	.	.	.	.	.	.	.	.	.	.	.	.	.	.	.	.	.	.	.	.	.	.	.	.	.	.	.	ACE1^{Br48}
PY6017	.	.	.	.	.	.	.	.	.	.	.	.	.	.	.	.	.	.	.	.	.	.	.	.	.	.	.	ACE1^{Br48}
PY6045	.	.	.	.	.	.	.	.	.	.	.	.	.	.	.	.	.	.	.	.	.	.	.	.	.	.	.	ACE1^{Br48}
T25	.	.	.	.	.	.	.	.	.	.	.	.	.	.	.	.	.	.	.	.	.	.	.	.	.	.	.	ACE1^{Br48}
Br118.2	.	.	.	.	.	.	.	N	A	T	T	Q	L	R	L	G	A	K	QE	A	T	V	Q	S	A	T	A	ACE1$^{Br118.2}$
Br130	.	.	.	.	.	.	.	N	A	T	T	Q	L	R	L	G	A	K	QE	A	T	V	Q	S	A	T	A	ACE1$^{Br118.2}$
T109	NP	L	P	I	C	E	V	N	A	T	T	Q	L	R	L	G	A	K	QE	A	T	V	Q	S	A	T	A	ACE1^{T109}
B71	NP	L	P	I	C	E	V	N	A	T	T	Q	L	R	L	G	A	K	QE	A	T	V	Q	S	A	T	A	ACE1^{T109}
BdBar	NP	L	P	I	C	E	V	N	A	T	T	Q	L	R	L	G	A	K	QE	A	T	V	Q	S	A	T	A	ACE1^{T109}
BdMeh	NP	L	P	I	C	E	V	N	A	T	T	Q	L	R	L	G	A	K	QE	A	T	V	Q	S	A	T	A	ACE1^{T109}

Supplementary Fig. 8. Amino acid substitutions in ACE1 alleles found in MoT isolates. a Schematic representation of the domain structure of ACE1 proteins. **b** Location of polymorphic amino acid residues in ACE1 of MoT isolates.

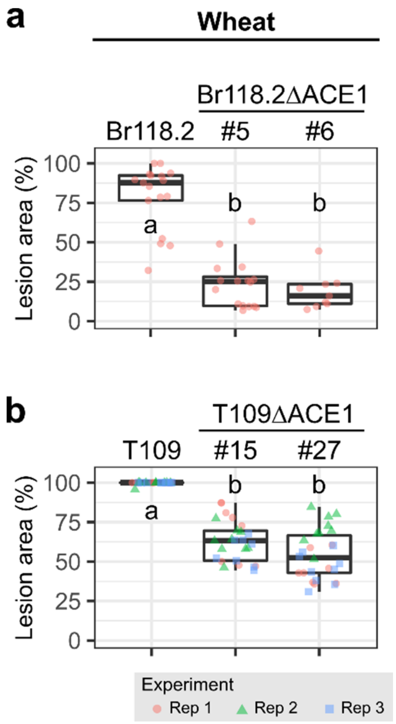

Supplementary Fig. 9. Quantitative evaluation of the effect of the disruption of *ACE1* alleles on aggressiveness of MoT isolates Br118.2 and T109. a Percentages of lesion area formed by MoT isolate Br118.2 and its *ACE1*-knockout mutants (Br118.2ΔACE1) on primary leaves of wheat cv. N4 (n = 17, 16, and 9 biologically independent samples). **b** Percentages of lesion area formed by MoT isolate T109 and its *ACE1*-knockout mutants (T109ΔACE1) on primary leaves of wheat cv. N4 (n = 27, 25, and 26 biologically independent samples). The boxplots show the percentage of lesion area from one or three independent experiments (seven to 17 plants per experiment per strain). Center lines show the medians; box limits indicate the 25th and 75th percentiles; whiskers extend to 1.5x the interquartile range from the 25th and 75th percentiles. Different letters indicate significant differences determined by Dunn's test at the 5 % level.

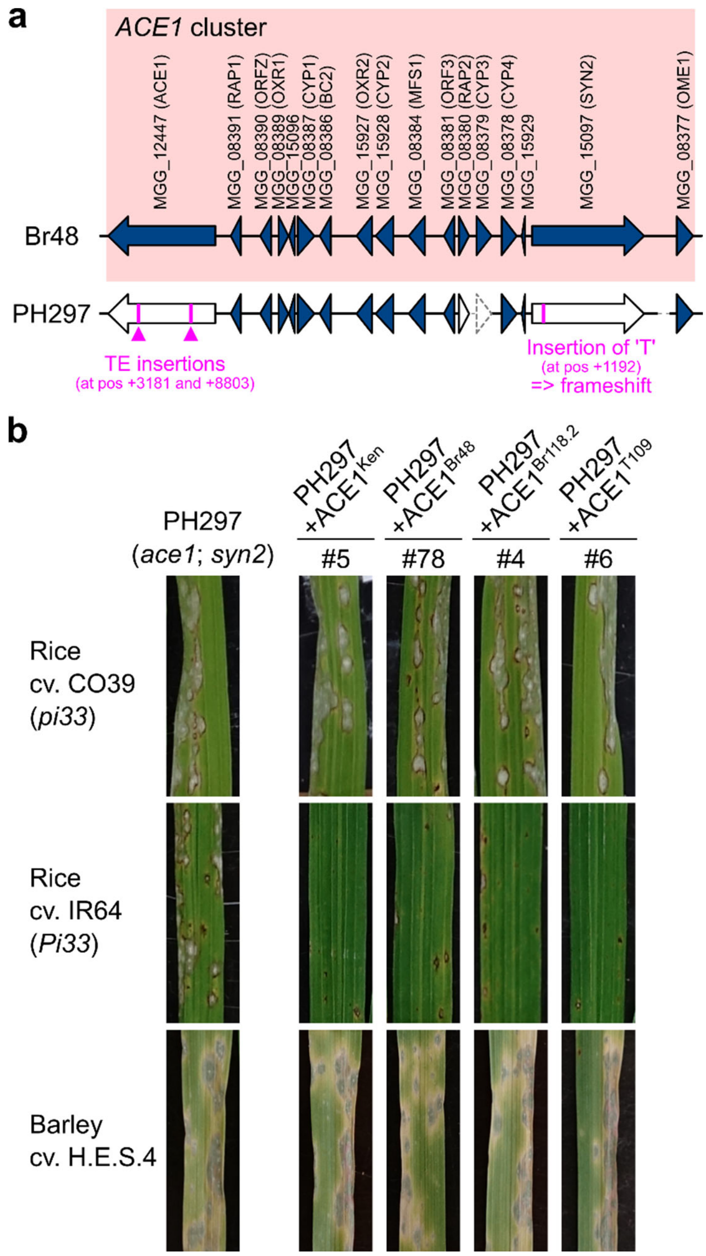

Supplementary Fig. 10. *SYN2* is not required for the function of *ACE1* alleles of MoT isolates as avirulence genes corresponding to *Pi33*. a Structure of the *ACE1* cluster in MoO isolate PH297. Both *ACE1* and *SYN2* are non-functional. **b** Reactions of rice and barley to transformants of PH297 carrying various *ACE1* alleles. Fourth leaves of rice cv. CO39 (*pi33*) and IR64 (*Pi33*) were inoculated with PH297, its transformants carrying *ACE1* of MoO isolate Ken53-33 (PH297+ACE1^{Ken}), and those carrying the *ACE1* alleles of MoT isolate Br48, Br118.2, and T109 (PH297+ACE1^{Br48}, PH297+ACE1$^{Br118.2}$, and PH297+ACE1^{T109}), and incubated at 26°C (rice)

or 22°C (barley) for 5 days. Similar results were obtained in two independent experiments (three to seven plants per experiment per strain).

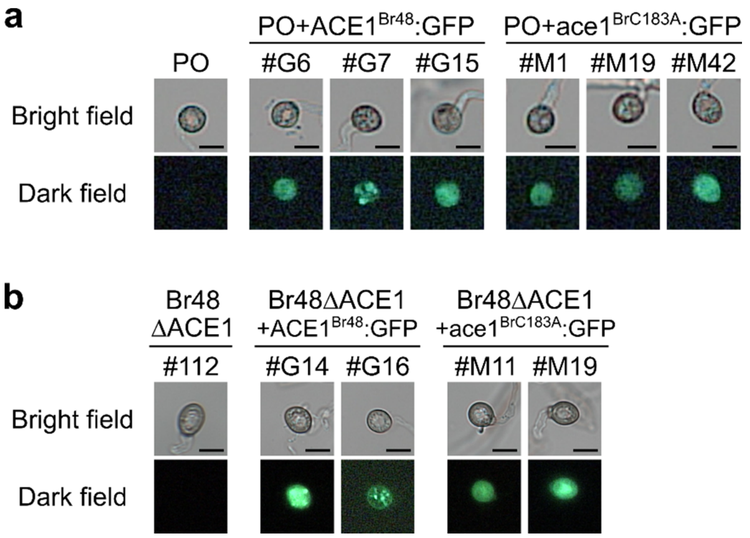

Supplementary Fig. 11. Microscopic observation of the expression of ACE1-GFP fusion proteins in appressoria of PO12-7301-2 and Br48 transformants. a Appressoria of MoO isolate PO12-7301-2 (PO), its transformants carrying intact *ACE1^{Br48}* fused to a GFP gene (PO+ACE1^{Br48}:GFP), and those carrying *ACE1^{Br48}* with the C183A mutation fused to a GFP gene (PO+ace1^{BrC183A}:GFP). **b** Appressoria of *ACE1*-knockout mutant of MoT isolate Br48 (Br48ΔACE1), and transformants of Br48ΔACE1 (#112) carrying intact *ACE1^{Br48}* fused to a GFP gene (Br48ΔACE1+ACE1^{Br48}:GFP), and those carrying *ACE1^{Br48}* with the C183A mutation fused to a GFP gene (Br48ΔACE1+ace1^{BrC183A}:GFP). Glass cover slips were inoculated with conidia, incubated at 25°C for 20h, and observed under bright and dark fields of a fluorescence microscope with an exciter filter B. Bars indicate 10 µm.

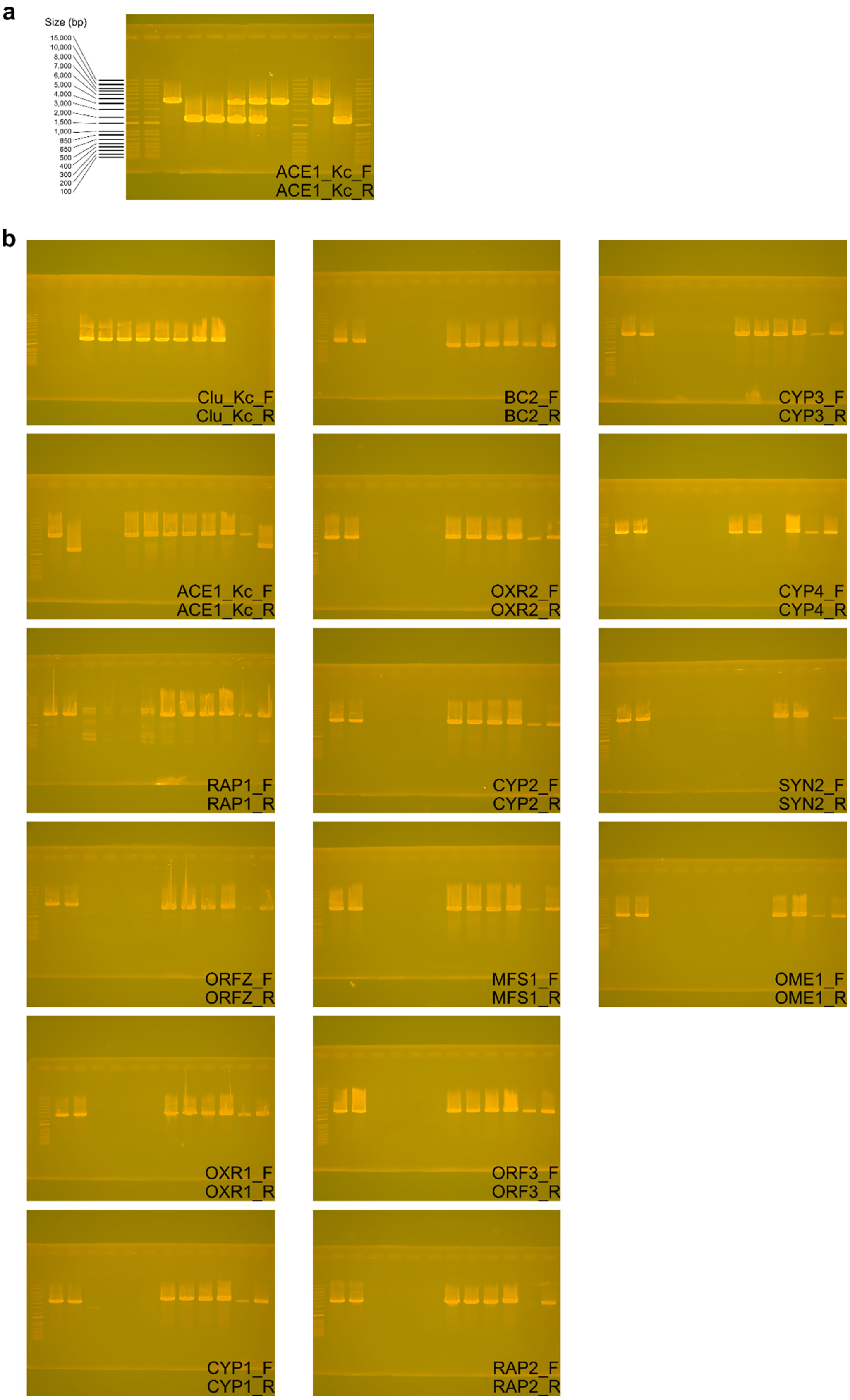
a
Size (bp)
15,000
10,000
8,000
7,000
6,000
5,000
4,000
3,000
2,000
1,500
1,000
850
650
500
400
300
200
100
ACE1_Kc_F
ACE1_Kc_R
b
Clu_Kc_F
Clu_Kc_R
BC2_F
BC2_R
CYP3_F
CYP3_R
ACE1_Kc_F
ACE1_Kc_R
OXR2_F
OXR2_R
CYP4_F
CYP4_R
RAP1_F
RAP1_R
CYP2_F
CYP2_R
SYN2_F
SYN2_R
ORFZ_F
ORFZ_R
MFS1_F
MFS1_R
OME1_F
OME1_R
OXR1_F
OXR1_R
ORF3_F
ORF3_R
CYP1_F
CYP1_R
RAP2_F
RAP2_R

Supplementary Fig. 12. Uncropped and unedited gel images. Original gel pictures for Supplementary Figure 2b (**a**) and Supplementary Figure 5b (**b**). For **a**, 3rd to 7th lanes are shown in Supplementary Figure 2b. For **b**, 2nd to 11th lanes are shown in Supplementary Figure 5b. Primer pairs used for PCR amplification are indicated on each gel. The same molecular weight ladder was used for all of the gels.

Supplementary Table 1. Reactions of representative wheat and barley cultivars to *P. oryzae* isolates and their progenies.

		Reactions[a] to parental strains[b]			No. of BC_1F_1 cultures derived from 54N1 x Br48			
Species	Cultivar	PO12-7301-2	54N1	Br48	G-	G+	Total	$\chi^2(1:1)$
Triticum aestivum	Norin 4 (N4)	B-	G-	G+	47	47	94	0
Hordeum vulgare	H.E.S.4	B-	G-	G+	47[c]	47[d]	94	0

[a] B-, discrete brown lesion; B+, confluent brown lesions; G-, discrete green lesions; G+, confluent green lesions (1).

[b] PO12-7301-2, MoO isolate; Br48, MoT isolate; 54N1, an F_1 culture derived from PO12-7301-2 x Br48.

[c] The 47 cultures that showed G- on N4.

[d] The 47 cultures that showed G+ on N4.

Supplementary Table 2. Fungal isolates used in this study.

Isolate	Species	Host	Year	Locality	Sequence source	Accession no.
PO12-7301-2	*Pyricularia oryzae*	*Oryza sativa*	1973	Tamangobo, Indonesia	This study	SAMD00549579
Ken53-33	*P. oryzae*	*O. sativa*	1953	Aichi, Japan	This study	SAMD00549578
PH297	*P. oryzae*	*O. sativa*	2012	Laguna, Philippines	This study	SAMD00549580
IN77-20-1-1	*P. oryzae*	*Setaria italica*	1977	Mysore, India	This study	SAMD00549586
MZ5-1-6	*P. oryzae*	*Eleusine coracana*	1976	Miyazaki, Japan	Luciano et al. 2019 (2)	SAMN10491321
TP2	*P. oryzae*	*Lolium perenne*	1997	Tochigi, Japan	Inoue et al. 2017 (3)	SAMD00069326
Br48	*P. oryzae*	*Triticum aestivum*	1990	Mato Grosso do Sul, Brazil	Yoshida et al. 2016 (4)	SAMD00051171
Br118.2	*P. oryzae*	*T. aestivum*	1992	Paraná, Brazil	Inoue et al. 2017 (3)	SAMD00069325
T109 (BTGP-6(e))	*P. oryzae*	*T. aestivum*	2017	Meherpur, Bangladesh	Soanes et al. 2017 (5)	SAMEA104190808

Supplementary Table 3. Plasmids used in this study.

Name	Description	Construction/Reference
pBlueScript II SK(+)	Standard cloning vector.	Agilent Technologies, CA, USA
pSH75	Fungal transformation vector containing a hygromycin resistance cassette as a selection marker.	Kimura and Tsuge 1993 (6)
pII99	Fungal transformation vector containing a geneticin resistance cassette as a selection marker.	Namiki et al. 2001 (7)
pEGFP75	A plasmid vector carrying eGFP gene under the control of the *Aspergillus nidulans trpC* promoter and terminator.	Kadotani et al. 2003 (8)
pCRISPR/Cas-U6-1	CRISPR/Cas9 vector for *P. oyrzae*. Codon-optimized Cas9 sequence and gRNA are driven by *Aureobasidium pullulans* translation elongation factor promoter and *P. oryzae* RNA polymerase III U6 promoter, respectively.	Arazoe et al. 2015 (9)
ZT3-8-D	A Br48 BAC clone containing a part of the *PWT2* candidate interval.	
ZT1-1-A	A Br48 BAC clone containing a part of the *PWT2* candidate interval.	
ZT3-2-D	A Br48 BAC clone containing the whole *ACE1* cluster.	
pACE1^{Ken}	pBlueScript II SK(+)-derived plasmid containing a ~15-kb EcoRI fragment of Ken53-33 encompassing the *ACE1^{Ken}* gene.	A ~1.6-kb upstream, ORF, and ~1.4-kb downstream fragments of *ACE1^{Ken}* was PCR-amplified from Ken53-33 gDNA with primer pairs ACE1_cU_F (5'-AATTCTCTTATAACCGATGATCTGTTC-3') and ACE1_cU_R (5'-TCCACATCTCGTCACGCAT-3'), ACE1_O_F1 (5'-ATGCGTGACGAGATGTGGAATAC-3') and ACE1_O_R2 (5'-TCAGTTCCCGTCCTTCAGCA-3'), and ACE1_cD_F (5'-CTGAAGGACGGGAACTGATTC-3') and ACE1_cD_R (5'-GAATTCGCAAGAGTGAGACAC-3'), respectively. Vector backbone of pBlueScript II SK(+) was PCR-amplified with primers pBS_F_E15k (5'-tcactcttgcgaattcATCGAATTCCTGCAGCC-3') and pBS_R_E15k (5'-ggttataagagaattcATCAAGCTTATCGATACCGTC-3'). The four fragments were assembled by InFusion reaction.
pACE1$^{Br118.2}$	pBlueScript II SK(+)-derived plasmid containing a ~15-kb EcoRI fragment of Br118.2 encompassing the *ACE1$^{Br118.2}$* gene.	A ~1.6-kb upstream, ORF, and ~1.4-kb downstream fragments of *ACE1$^{Br118.2}$* was PCR-amplified from Br118.2 gDNA with primer pairs ACE1_cU_F and ACE1_cU_R, ACE1_O_F1 and ACE1_O_R2, and ACE1_cD_F and ACE1_cD_R, respectively. Vector backbone of pBlueScript II SK(+) was PCR-amplified with primers pBS_F_E15k and pBS_R_E15k. The four fragments were assembled by InFusion reaction.
pACE1^{T109}	pBlueScript II SK(+)-derived plasmid containing a ~15-kb EcoRI fragment of T109 encompassing the *ACE1^{T109}* gene.	A ~1.6-kb upstream, ORF, and ~1.4-kb downstream fragments of *ACE1^{T109}* was PCR-amplified from T109 gDNA with primer pairs ACE1_cUM_F (5'-AATTCTCTTATAACCGATCATCTGTTC-3') and ACE1_cU_R, ACE1_O_F1 and ACE1_O_R2, and ACE1_cD_F and ACE1_cD_R, respectively. Vector backbone of pBlueScript II SK(+) was PCR-amplified with primers pBS_F_E15k and pBS_R_E15k. The four fragments were assembled by InFusion reaction.
pACE1^{Br48}	pBlueScript II SK(+)-derived plasmid containing a ~15-kb EcoRI fragment of Br48 encompassing the *ACE1^{Br48}* gene.	A ~15-kb EcoRI restriction fragment containing *ACE1^{Br48}* was liberated from a Br48 BAC clone ZA1-1-A. Vector backbone of pBlueScript II SK(+) was PCR-amplified with primers pBS_F_E15k and pBS_R_E15k. The two fragments were assembled by InFusion reaction.
pACE1^{Br48}-GFP	pACE1^{Br48}-derived plasmid containing the intact ACE1^{Br48}, C-terminally fused with a GFP gene.	A 16.4-kb NotI fragment containing ~1.6-kb upstream and N-terminal part of the ORF sequences of *ACE1^{Br48}* and the vector backbone of pBlueScript II SK(+) was liberated from pACE1^{Br48}. The C-terminal part of the *ACE1^{Br48}* ORF was PCR-amplified from pACE1^{Br48} with primers pACE1_N_F (5'-CGACCCCAGAGGCATGAG-3') and ACE1woStop_R (5'-cgaaccgcctccgctaccgccGTTCCCGTCCTTCAGCAG-3'). A GFP gene fragment was PCR-amplified from pEGFP75 with primers GFP_i_F (5'-agcggaggcggttcgggaATGGTGAGCAAGGGCGA-3') and GFP_i_R (5'-tccaaagttcatgaaTTACTTGTACAGCTCGTCCA-3'). The downstream region of *ACE1^{Br48}* gene including the putative terminator sequence was PCR-amplified from pACE1^{Br48} with primers ACE1ter_F (5'-TTCATGAACTTTGGAATACTGT-3') and pACE1_N_R (5'-AGGGAACAAAAGCTGGAG-3'). The four fragments were assembled by InFusion reaction.
pACE1^{BrC183A}-GFP	pACE1^{Br48}-GFP-derived plasmid containing the	A 53-bp DNA fragment carrying 'TGC' to 'GCT' mutation in the codon position for the 183rd amino acid of ACE1^{Br48} was generated by annealing two oligos, F_C183A (5'-

	ACE1^{Br48} with the C183A mutation, C-terminally fused with a GFP gene.	CCCTCGGTCACGGTCGATACCGCAGCTTCTTCCAGCTTGGTGGCCGTCCACCA-3') and R_C183A (5'-TGGTGGACGGCCACCAAGCTGGAAGAAGCTGCGGTATCGACCGTGACCGAGGG-3'). The DNA fragment was used to replace the corresponding sequence in pACE1^{Br48}-GFP by InFusion reaction with an ~18.6-kb BspEI/FseI double-digestion fragment liberated from pACE1^{Br48}-GFP and a ~0.4-kb upstream and a ~0.2-kb downstream flanking sequences of the mutation target site which were PCR-amplified from pACE1^{Br48}-GFP with primers ACE1_m_F1 (5'-CGTTGACAAATACTTTCATCCGG-3') and ACE1_m_R1 (5'-GTATCGACCGTGACC-3') and ACE1_m_F2 (5'-AGCTTGGTGGCCGTCC-3') and ACE1_m_R2 (5'-CATCCCACATGCGCGAG-3'), respectively.
pKO_ACE1	pACE1^{Br48}-derived plasmid containing an *ACE1* deletion construct which consists of the *ACE1*^{Br48} gene disrupted by a hygromycin resistance cassette placed in the middle of the gene.	A ~3.6-kb AgeI fragment of pACE1^{Br48} was replaced with the hygromycin resistance cassette. A hygromycin resistance cassette was PCR-amplified from pSH75 with primers HygR_F2_pACE1 (5'-aacatgctcaaccggtGTCGACAGAAGATGATATTGAAGG-3') and HygR_R2_pACE1 (5'-gagggaagccaccggtGGATCCGGTCGGCATCTAC-3') and assembled via InFusion reaction with a 14.7-kb AgeI fragment liberated from pACE1^{Br48}.
pSYN2^{Br48}	pBlueScript II SK(+)-derived plasmid containing a ~16-kb fragment of Br48 encompassing the *SYN2*^{Br48} gene.	A 14.3-kb DraI fragment containing *SYN2*^{Br48} ORF and ~1.4-kb downstream region was liberated from a Br48 BAC clone ZT3-2-D. A ~1.7-kb upstream fragment was PCR-amplified with primers SYN2_cU_F (5'-TGGATATTTACCGTGACGAAGA-3') and SYN2_cU_R (5'-ACCTGAAGTTTGATGGTGAACA-3'). Vector backbone of pBlueScript II SK(+) was PCR-amplified with primers pBS_F_DS (5'-aaaaaaagtaggtttaaaATCGAATTCCTGCAGCC-3') and pBS_R_wU (5'-cacggtaaatatccaATCAAGCTTATCGATACCGTC-3'). The three fragments were assembled by InFusion reaction.
pKO_SYN2	pBlueScript II SK(+)-derived plasmid containing a *SYN2* deletion construct which consists of a hygromycin resistance cassette flanked by the left and right borders of the *SYN2*^{Br48} gene.	The left and right borders of *SYN2*^{Br48} were PCR-amplified from Br48 gDNA with primers SYN2_Ku_F (5'-atcgataagcttgatCCGGGAGATGGAGACGTTTA-3') and SYN2_Ku_R (5'-tcatcttctgtcgacGCCTAAAGAGTGTCCGAGGT-3') and SYN2_Kd_F (5'-atgccgaccggatccCGCTGTGGTGGGAAAAGATT-3') and SYN2_Kd_R (5'-ctgcaggaattcgatCCTGACCCTCACCCAAATCT-3'), respectively. The hygromycin resistance cassette was PCR-amplified from pSH75 with primers HygR_F2 (5'-GTCGACAGAAGATGATATTGAAGG-3') and HygR_R2 (5'-GGATCCGGTCGGCATCTAC-3'). The three fragments were integrated via InFusion reaction into EcoRV-linearized vector pBlueScript II SK(+).
pKO_ACE1clu	pBlueScript II SK(+)-derived plasmid containing an *ACE1* cluster deletion construct which consists of a hygromycin resistance cassette flanked by the left and right borders of the *ACE1* cluster of Br48.	The left and right borders of Br48 *ACE1* cluster were PCR-amplified from Br48 gDNA with primers Clu_Kl_F (5'-atcgataagcttgatGGCACAACTACAGAGGGCTA-3') and Clu_Kl_R (5'-tcatcttctgtcgacAGCCTGTACAGCAAGACCAT-3') and Clu_Kr_F (5'-atgccgaccggatccGACTTGTACGTCGGCTACA-3') and Clu_Kr_R (5'-ctgcaggaattcgatCGTACGTAAACCCCAACCT-3'), respectively. A hygromycin resistance cassette was PCR-amplified from pSH75 with primers HygR_F2 and HygR_R2. The three fragments were integrated via InFusion reaction into EcoRV-linearized vector pBlueScript II SK(+).
pCRISPR_ACE1	pCRISPR/Cas-U6-1-derived plasmid containing a gRNA sequence targeting *ACE1*.	A fragment containing a guide sequence targeting *ACE1*, generated by annealing two oligos ACE1_gRNA_F (5'-ttcgAGGACCTCGCCCGCATGGT-3') and ACE1_gRNA_R (5'-aaacACCATGCGGGCGAGGTCCT-3'), was integrated via Golden Gate reaction into *Esp*3I-linearized vector pCRISPR/Cas-U6-1.
pCRISPR_SYN2	pCRISPR/Cas-U6-1-derived plasmid containing a gRNA sequence targeting *SYN2*.	A fragment containing a guide sequence targeting *SYN2*, generated by annealing two oligos SYN2_gRNA_F (5'-ttcgCAACAAATACCCCGCGCTC-3') and SYN2_gRNA_R (5'-aaacGAGCGCGGGGTATTTGTTG-3'), was integrated via Golden Gate reaction into *Esp*3I-linearized vector pCRISPR/Cas-U6-1.
pCRISPR_ACE1clu	pCRISPR/Cas-U6-1-derived plasmid containing a gRNA sequence targeting *ORF3* within the *ACE1* cluster.	A fragment containing a guide sequence targeting *ORF3*, generated by annealing two oligos Clu_gRNA_F (5'-ttcgTACTCGCCCAGAAGACGTA-3') and Clu_gRNA_R (5'-aaacTACGTCTTCTGGGCGAGTA-3'), was integrated via Golden Gate reaction into *Esp*3I-linearized vector pCRISPR/Cas-U6-1.

Supplementary Table 4. *P. oryzae* strains generated in this study.

Strain	Description	Plasmid introduced
54N1 + EV	54N1 transformant carrying an empty vector	pBlueScript II SK(+), pSH75
54N1 + pACE1^{Br48}	54N1 transformant carrying a ~15-kb genomic fragment of Br48 containing *ACE1^{Br48}*	pACE1^{Br48}, pSH75
54N1 + ZT3-8-D	54N1 transformant carrying a Br48 BAC clone containing the part of *PWT2* candidate interval	ZT3-8-D, pSH75
54N1 + ZT1-1-A	54N1 transformant carrying a Br48 BAC clone containing the part of *PWT2* candidate interval	ZT1-1-A, pSH75
Br48ΔACE1	*ACE1* KO mutant of Br48	pKO_ACE1
Br48ΔACE1 + ACE1^{Br48}	Br48ΔACE1 transformant harboring genomic fragment encompassing *ACE1^{Br48}*	pACE1^{Br48}, pII99
Br48ΔACE1 + ACE1^{Br48}:GFP	Br48ΔACE1 transformant carrying intact *ACE1^{Br48}* fused to a GFP gene	pACE1^{Br48}-GFP, pII99
Br48ΔACE1 + ace1^{BrC183A}:GFP	Br48ΔACE1 transformant carrying *ACE1^{Br48}* with the C183A mutation fused to a GFP gene	pACE1^{BrC183A}-GFP, pII99
Br48ΔSYN2	*SYN2* KO mutant of Br48	pKO_SYN2, pCRISPR_SYN2
Br48ΔSYN2 + SYN2^{Br48}	Br48ΔSYN2 transformant harboring genomic fragment encompassing *SYN2^{Br48}*	pSYN2^{Br48}, pII99
Br48ΔACE1cluster	*ACE1* cluster KO mutant of Br48	pKO_ACE1clu, pCRISPR_ACE1clu
Br48ΔACE1cluster + ACE1^{Br48}	Br48ΔACE1cluster transformant harboring genomic fragment encompassing *ACE1^{Br48}*	pACE1^{Br48}, pII99
Br48ΔACE1cluster + ZT1-1-A	Br48ΔACE1cluster transformant harboring a genomic fragment containing a partial *ACE1* cluster (lacking a part of *SYN2* and the entire *OME1*) of Br48	ZT1-1-A, pII99
Br48ΔACE1cluster + ZT3-2-D	Br48ΔACE1cluster transformant harboring a genomic fragment containing the whole *ACE1* cluster of Br48	ZT3-2-D, pII99
KenΔACE1	*ACE1* KO mutant of Ken53-33	pKO_ACE1, pCRISPR_ACE1
INΔACE1	*ACE1* KO mutant of IN77-20-1-1	pKO_ACE1, pCRISPR_ACE1
MZΔACE1	*ACE1* KO mutant of MZ5-1-6	pKO_ACE1, pCRISPR_ACE1
TP2ΔACE1	*ACE1* KO mutant of TP2	pKO_ACE1, pCRISPR_ACE1
Br118.2ΔACE1	*ACE1* KO mutant of Br118.2	pKO_ACE1, pCRISPR_ACE1
T109ΔACE1	*ACE1* KO mutant of T109	pKO_ACE1, pCRISPR_ACE1
PO + EV	PO12-7301-2 transformant carrying an empty vector	pBlueScript II SK(+), pSH75
PO + ACE1^{Ken}	PO12-7301-2 transformant harboring a genomic fragment encompassing *ACE1^{Ken}*	pACE1^{Ken}, pSH75
PO + ACE1^{Br48}	PO12-7301-2 transformant harboring a genomic fragment encompassing *ACE1^{Br48}*	pACE1^{Br48}, pSH75
PO + ACE1$^{Br118.2}$	PO12-7301-2 transformant harboring a genomic fragment encompassing *ACE1$^{Br118.2}$*	pACE1$^{Br118.2}$, pSH75
PO + ACE1^{T109}	PO12-7301-2 transformant harboring a genomic fragment encompassing *ACE1^{T109}*	pACE1^{T109}, pSH75
PO + ACE1^{Br48}:GFP	PO12-7301-2 transformant carrying intact *ACE1^{Br48}* fused to a GFP gene	pACE1^{Br48}-GFP, pSH75
PO + ace1^{BrC183A}:GFP	PO12-7301-2 transformant carrying *ACE1^{Br48}* with the C183A mutation fused to a GFP gene	pACE1^{BrC183A}-GFP, pSH75
PH297 + ACE1^{Ken}	PH297 transformant harboring a genomic fragment encompassing *ACE1^{Ken}*	pACE1^{Ken}, pSH75
PH297 + ACE1^{Br48}	PH297 transformant harboring a genomic fragment encompassing *ACE1^{Br48}*	pACE1^{Br48}, pSH75
PH297 + ACE1$^{Br118.2}$	PH297 transformant harboring a genomic fragment encompassing *ACE1$^{Br118.2}$*	pACE1$^{Br118.2}$, pSH75
PH297 + ACE1^{T109}	PH297 transformant harboring a genomic fragment encompassing *ACE1^{T109}*	pACE1^{T109}, pSH75

Supplementary Table 5. Fungal genome sequence data used in this study.

Isolate ID	Species	Host	Year	Locality	Sequence source	Accession no.
Ina168	*Pyricularia oryzae*	*Oryza sativa*	1958	Aichi, Japan	Yoshida et al. 2016 (4)	SAMD00051169
Y34	*P. oryzae*	*Oryza sativa*	1982	Yunnan, China	Xue et al. 2012 (10)	SAMN02981398
Guy11	*P. oryzae*	*Oryza sativa*	1988	French Guyana	Bao et al. 2017 (11)	SAMN06050153
FR13	*P. oryzae*	*Oryza sativa*	1988	France	Chiapello et al. 2015 (12)	SAMEA3231509
PH14	*P. oryzae*	*Oryza sativa*	ND	Philippines	Chiapello et al. 2015 (12)	SAMEA3231517
GFSI1-7-2	*P. oryzae*	*Setaria italica*	1977	Gifu, Japan	Yoshida et al. 2016 (4)	SAMD00051170
GrF52	*P. oryzae*	*Setaria viridis*	2001	Kentucky, U.S.A.	Gladieux et al. 2018 (13)	SAMN08009556
Sv9623	*P. oryzae*	*Setaria viridis*	1996	Zhejian, China	Zhong et al. 2016 (14)	SAMN04318450
Sv9610	*P. oryzae*	*Setaria viridis*	1996	Zhejian, China	Zhong et al. 2016 (14)	SAMN04318449
US71	*P. oryzae*	*Setaria* spp.	ND	U.S.A.	Chiapello et al. 2015 (12)	SAMEA3232119
Z2-1	*P. oryzae*	*Eleusine coracana*	1977	Kagawa, Japan	Yoshida et al. 2016 (4)	SAMD00051173
G22	*P. oryzae*	*Eleusine coracana*	1976	Japan	Gladieux et al. 2018 (13)	SAMN08009554
EI9411	*P. oryzae*	*Eleusine indica*	1994	Fujian, China	Zhong et al. 2016 (14)	SAMN04318447
CD156	*P. oryzae*	*Eleusine indica*	1989	Ferkessedougou, Ivory Coast	Chiapello et al. 2015 (12)	SAMEA3231215
EI9604	*P. oryzae*	*Eleusine indica*	1996	Zhejiang, China	Zhong et al. 2016 (14)	SAMN04318448
BR62	*P. oryzae*	*Eleusine indica*	1991	Brazil	Islam et al. 2016 (15)	SAMEA4029901
B51	*P. oryzae*	*Eleusine indica*	2012	Quirusillas, Bolivia	Pieck et al. 2017 (16)	SAMN08009542
PH42	*P. oryzae*	*Eleusine indica*	1983	Philippines	Farman et al. 2017 (17)	SAMN08009570
LpKY97	*P. oryzae*	*Lolium perenne*	1997	Kentucky, U.S.A.	Rahnama et al. 2020 (18)	SAMN14533965
PgKY	*P. oryzae*	*Lolium perenne*	2000	Kentucky, U.S.A.	Islam et al. 2016 (15)	SAMEA4029903
PGPA	*P. oryzae*	*Lolium perenne*	1998	Pennsylvania, U.S.A	Islam et al. 2016 (15)	SAMEA4029904
TP1	*P. oryzae*	*Lolium perenne*	1997	Tochigi, Japan	Asuke et al. 2020 (19)	SAMD00549600
CHRF	*P. oryzae*	*Lolium perenne*	1996	Maryland, U.S.A.	Pieck et al. 2017 (16)	SAMN08009548
CHW	*P. oryzae*	*Lolium perenne*	1996	Maryland, U.S.A.	Pieck et al. 2017 (16)	SAMN08009549
FH	*P. oryzae*	*Lolium perenne*	1997	Maryland, U.S.A.	Farman et al. 2017 (17)	SAMN08009551
GG11	*P. oryzae*	*Lolium perenne*	1997	Kentucky, U.S.A.	Farman et al. 2017 (17)	SAMN08009555
HO	*P. oryzae*	*Lolium perenne*	1996	Pennsylvania, U.S.A.	Farman et al. 2017 (17)	SAMN08009558
Br116.5	*P. oryzae*	*Triticum aestivum*	1992	Paraná, Brazil	Inoue et al. 2017 (3)	SAMD00069324
BdBar	*P. oryzae*	*Triticum aestivum*	2016	Barisal, Bangladesh	Malaker et al. 2016 (20)	SAMN04940126
BdMeh	*P. oryzae*	*Triticum aestivum*	2016	Meherpur, Bangladesh	Malaker et al. 2016 (20)	SAMN04942534
B2	*P. oryzae*	*Triticum aestivum*	2011	Bolivia	Pieck et al. 2017 (16)	SAMN05580113
B71	*P. oryzae*	*Triticum aestivum*	2012	Bolivia	Malaker et al. 2016 (20)	SAMN04942725
Br7	*P. oryzae*	*Triticum aestivum*	1990	Paraná, Brazil	Pieck et al. 2017 (16)	SAMN08009545

BR32	*P. oryzae*	*Triticum aestivum*	1991	Brazil	Chiapello et al. 2015 (12)	SAMEA3231213
Br80	*P. oryzae*	*Triticum aestivum*	1991	Brazil	Pieck et al. 2017 (16)	SAMN08009546
Br130	*P. oryzae*	*Triticum aestivum*	1990	Mato Grosso do Sul, Brazil	Farman et al. 2017 (17)	SAMN08009547
PY0925	*P. oryzae*	*Triticum aestivum*	2009	Minas Gerais, Brazil	Islam et al. 2016 (15)	SAMEA4029894
PY36.1	*P. oryzae*	*Triticum aestivum*	2007	Brasília, Brazil	Islam et al. 2016 (15)	SAMEA4029897
PY5033	*P. oryzae*	*Triticum aestivum*	2005	Paraná, Brazil	Islam et al. 2016 (15)	SAMEA4029889
PY6017	*P. oryzae*	*Triticum aestivum*	2006	Minas Gerais, Brazil	Islam et al. 2016 (15)	SAMEA4029890
PY6045	*P. oryzae*	*Triticum aestivum*	2006	Goiás, Brazil	Islam et al. 2016 (15)	SAMEA4029900
T25	*P. oryzae*	*Triticum aestivum*	1988	Paraná, Brazil	Pieck et al. 2017 (16)	SAMN08009575
P3	*P. oryzae*	*Triticum durum*	2012	Canindeyu, Paraguay	Pieck et al. 2017 (16)	SAMN08009568
Dig41	*P. grisea*	*Digitaria sanguinalis*	1990	Hyogo, Japan	Yoshida et al. 2016 (4)	SAMD00051174

Supplementary References

1. Tosa, Y., Tamba, H., Tanaka, K. & Mayama, S. Genetic analysis of host species specificity of *Magnaporthe oryzae* isolates from rice and wheat. *Phytopathology* **96**, 480–484 (2006).
2. Gómez Luciano, L. B. et al. Blast fungal genomes show frequent chromosomal changes, gene gains and losses, and effector gene turnover. *Mol. Biol. Evol.* **36**, 1148–1161 (2019).
3. Inoue, Y. et al. Evolution of the wheat blast fungus through functional losses in a host specificity determinant. *Science* **357**, 80–83 (2017).
4. Yoshida, K. et al. Host specialization of the blast fungus *Magnaporthe oryzae* is associated with dynamic gain and loss of genes linked to transposable elements. *BMC Genom.* **17**, 370 (2016).
5. Soanes, D., Ryder, L. S., Islam, M. T. & Talbot, N. J. Data from "Genome assemblies of *Magnaporthe oryzae* isolated from Bangladesh in 2016 and 2017." Figshare. Available at https://doi.org/10.6084/m9.figshare.5236381.v1. Deposited 11 November 2017.
6. Kimura, N. & Tsuge, T. Gene cluster involved in melanin biosynthesis of the filamentous fungus *Alternaria alternata*. *J. Bacteriol.* **175**, 4427–4435 (1993).
7. Namiki, F. et al. Mutation of an arginine biosynthesis gene causes reduced pathogenicity in *Fusarium oxysporum* f. sp. *melonis*. *Mol. Plant-Microbe Interact.* **14**, 580–584 (2001).
8. Kadotani, N., Nakayashiki, H., Tosa, Y. & Mayama, S. RNA silencing in the phytopathogenic fungus *Magnaporthe oryzae*. *Mol. Plant-Microbe Interact.* **16**, 769–776 (2003).
9. Arazoe, T. et al. Tailor-made CRISPR/Cas system for highly efficient targeted gene replacement in the rice blast fungus. *Biotechnol. Bioeng.* **112**, 2543–2549 (2015).
10. Xue, M. et al. Comparative analysis of the genomes of two field isolates of the rice blast fungus *Magnaporthe oryzae*. *PLoS Genet.* **8**, e1002869 (2012).
11. Bao, J. et al. PacBio sequencing reveals transposable elements as a key contributor to genomic plasticity and virulence variation in *Magnaporthe oryzae*. *Mol. Plant* **10**, 1465–1468 (2017).
12. Chiapello, H. et al. Deciphering genome content and evolutionary relationships of isolates from the fungus *Magnaporthe oryzae* attacking different host plants. *Genome Biol. Evol.* **7**, 2896–2912 (2015).
13. Gladieux, P. et al. Gene flow between divergent cereal- and grass-specific lineages of the rice blast fungus *Magnaporthe oryzae*. *mBio* **9**, e01219-17 (2018).
14. Zhong, Z. et al. Directional selection from host plants is a major force driving host specificity in *Magnaporthe* species. *Sci. Rep.* **6**, 25591 (2016).

15. Islam, M. T. et al. Emergence of wheat blast in Bangladesh was caused by a South American lineage of *Magnaporthe oryzae*. *BMC Biol.* **14**, 84 (2016).
16. Pieck, M. L. et al. Genomics-based marker discovery and diagnostic assay development for wheat blast. *Plant Dis.* **101**, 103–109 (2017).
17. Farman, M. et al. The *Lolium* pathotype of *Magnaporthe oryzae* recovered from a single blasted wheat plant in the United States. *Plant Dis.* **101**, 684–692 (2017).
18. Rahnama, M. et al. Transposon-mediated telomere destabilization: a driver of genome evolution in the blast fungus. *Nucleic Acids Res.* **48**, 7197–7217 (2020).
19. Asuke, S. et al. Evolution of an *Eleusine*-specific subgroup of *Pyricularia oryzae* through a gain of an avirulence gene. *Mol. Plant-Microbe Interact.* **33**, 153–165 (2020).
20. Malaker, P. K. et al. First report of wheat blast caused by *Magnaporthe oryzae* pathotype *triticum* in Bangladesh. *Plant Dis.* **100**, 2330–2330 (2016).
